# Algorithmic bias in HR recruitment systems: A qualitative analysis of managerial risk and sociological implications

**DOI:** 10.1371/journal.pone.0349400

**Published:** 2026-06-03

**Authors:** Baijia Song

**Affiliations:** Shandong Vocational and Technical University of International Studies‌, Rizhao, China; Wroclaw University of Economics and Business: Uniwersytet Ekonomiczny we Wroclawiu, POLAND

## Abstract

Algorithmic hiring systems have rapidly proliferated across industries, promising improved efficiency, objectivity, and scalability in recruitment processes. However, growing empirical evidence reveals a gap between these expected benefits and actual outcomes, as many systems inadvertently reproduce or amplify historical inequalities embedded in training data. The main aim of this study is to develop and evaluate a rigorous, multi-layered framework capable of identifying, interpreting, and mitigating bias throughout the full lifecycle of algorithmic hiring systems, ensuring both immediate decision fairness and long-term career equity. To achieve this aim, the Multi-Layer Bias Analysis and Mitigation System (ML-BAMS) is introduced as a comprehensive approach to detecting and mitigating bias in HR recruitment systems. The framework integrates modules for bias decomposition and data diagnostics, recruitment-aware fairness evaluation across multi-stage pipelines, interpretability of opaque models through influence mapping, fairness-preserving representation learning, and longitudinal simulation of career mobility outcomes. Using synthetic hiring datasets, the proposed framework demonstrates substantial reductions in demographic disparities across key fairness metrics, including improvements in demographic parity (32.4%), equal opportunity (28.7%), equalized odds (25.9%), and treatment equality (19.2%), while maintaining competitive predictive accuracy (ΔAccuracy: + 0.024). These findings highlight the importance of integrated sociotechnical approaches that address bias transmission, enhance transparency, and account for long-term impacts. The ML-BAMS framework provides a practical and modular toolset for implementing responsible AI in recruitment, balancing operational performance with ethical considerations of fairness and social equity.

## 1. Introduction

Algorithmic hiring systems have rapidly become embedded within modern recruitment, selection, and promotion processes across nearly every industry [1]. Organizations increasingly rely on these systems to manage expanding applicant pools, accelerate screening procedures, and improve the consistency of hiring decisions [2]. The appeal is comapelling: automated tools can process thousands of applications in seconds, identify patterns that humans might overlook, and provide standardized scoring across candidates [3]. Algorithmic recruitment represents a transformative shift toward data-driven human resource management, offering the promise of objectivity, speed, and scalability. Yet the reality is far more complex [4]. As widespread implementation advances, a growing collection of internal audits, media investigations, organizational experiences, and empirical analyses reveal significant discrepancies between the idealized benefits of algorithmic hiring and its actual performance [5]. Rather than eliminating inequity, many systems have exhibited tendencies to reproduce, amplify, or introduce harmful forms of discrimination. These failures often stem from the very foundations upon which automated models are built historical data reflecting unequal labor markets, biased organizational practices, and broader societal inequalities [6]. When these patterns are encoded into training datasets or optimization objectives, algorithmic tools can inadvertently learn to penalize certain demographic groups, favor specific educational or career trajectories, or recognize proxies for sensitive attributes such as gender, race, or socioeconomic status [7]. The rapid rise of high-profile failures has intensified public awareness of the risks associated with algorithmic recruitment. Several major organizations have publicly acknowledged significant bias issues in their automated hiring tools, prompting widespread scrutiny, academic debate, and regulatory attention. These events illustrate how quickly algorithmic bias can escalate into reputational damage, legal exposure, and operational disruption [8].

**Fig 1:**
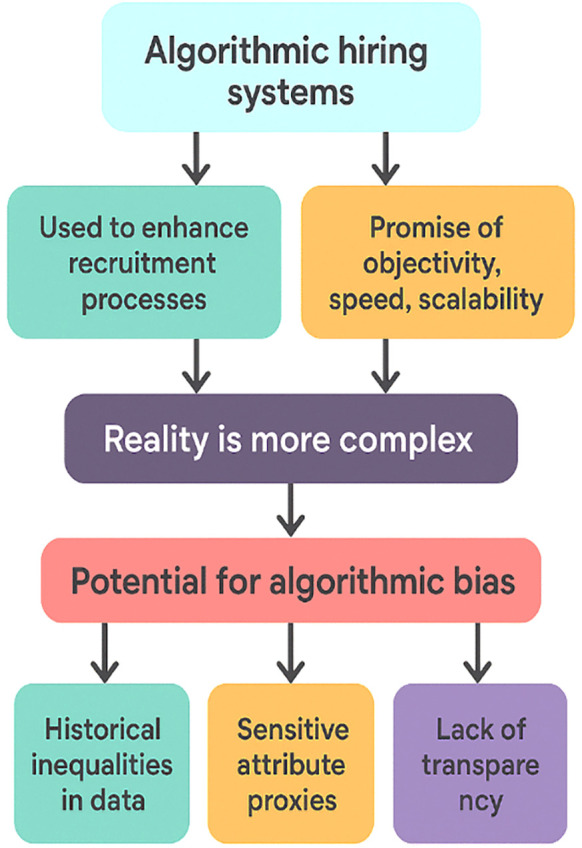
Overview of Key Dynamics in Algorithmic Hiring Systems.

Policymakers and governing bodies have started to respond by exploring regulatory frameworks that classify recruitment algorithms as high-risk decision-making systems, requiring heightened oversight, transparency, and accountability [9]. These developments reflect a broader recognition that hiring is not merely a technical process, but a deeply sociopolitical one with profound implications for economic opportunity and social mobility [10]. Within this evolving landscape, multiple stakeholders influence and are influenced by algorithmic hiring practices. Candidates are the most visibly affected group, often subjected to automated evaluations without clear explanations or avenues for appeal [11]. Vendors and software developers play a central role in shaping model architectures, selecting training data, and embedding value-laden assumptions into algorithmic logic [12]. Fig 1 presents a structured flowchart summarizing the core issues surrounding the use of algorithmic hiring systems. It begins by highlighting the intended purpose of these systems enhancing recruitment processes and delivering objectivity, speed, and scalability. The diagram then emphasizes that the practical reality is more complex than these promises suggest. As the flow progresses, it identifies the central challenge: the potential for algorithmic bias. Three major sources of such bias are outlined at the bottom of the figure historical inequalities embedded in training data, the presence of proxy variables that indirectly encode sensitive attributes, and the overall lack of transparency in algorithmic decision-making. Together, the components visually illustrate how well-intentioned automation can unintentionally reproduce unfair outcomes in recruitment contexts [13]. Employers deploying these tools must balance the desire for efficiency with the responsibility to ensure fair outcomes, maintain compliance, and protect their organizational reputation. Meanwhile, the algorithms themselves act as sociotechnical agents that mediate between candidates and employers, shaping access to opportunities across entire labor markets [14].

**Fig 2:**
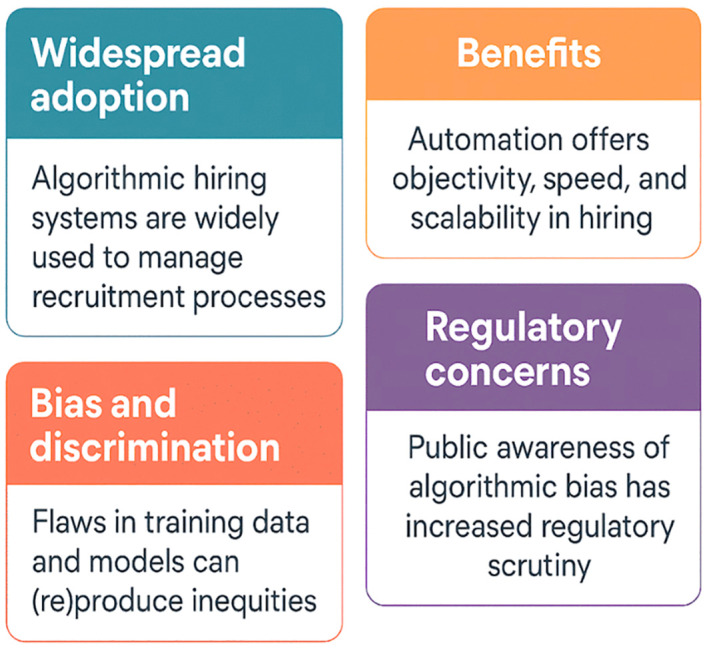
Key Dimensions of Algorithmic Hiring in Modern Recruitment.

As these systems grow in prevalence, they not only reflect organizational priorities but actively reshape the broader structure of employment pathways [15]. Central to the debate is the concept of fairness and its relationship to algorithmic bias. Fairness implies impartial treatment and the absence of unjustified distinctions between individuals. Bias, in contrast, refers to systematic deviations that produce unfair outcomes, whether through discriminatory treatment or indirect exclusion [16–19]. Algorithmic discrimination can manifest in explicit forms, such as penalizing candidates based on sensitive characteristics, or in implicit forms, such as penalizing characteristics statistically associated with those traits. Even when unintended, these distortions compromise the integrity of hiring decisions and undermine public trust in organizational practices [20–23]. The rise of advanced AI techniques introduces additional challenges. Modern systems rely on high-dimensional data, complex neural architectures, and automated feature extraction processes that can generate latent representations of candidate attributes beyond human interpretation. Such opacity becomes particularly troubling when algorithms are used for consequential decisions like hiring, where fairness, transparency, and accountability are essential public values. Social discourse increasingly reflects discomfort with reliance on opaque automated systems to determine access to employment, fueling concerns about hidden bias and unchecked algorithmic authority [ 24–26]. Fig 2 provides a concise, quadrant-style overview of the major factors shaping the use of algorithmic hiring systems. The top-left panel highlights their widespread adoption, noting that organizations increasingly rely on automated tools to streamline recruitment processes. The top-right panel outlines key benefits, including enhanced objectivity, operational speed, and scalability. The bottom-left panel addresses the risks of bias and discrimination, emphasizing how flawed or unequal training data can reproduce inequities in hiring outcomes [27].

The primary aim of this paper is to develop, formalize, and empirically validate a rigorous multi-layered framework capable of detecting, analyzing, and mitigating algorithmic bias across the entire lifecycle of AI-driven hiring systems. While existing approaches typically address isolated aspects of fairness or rely on post-hoc adjustments, this study seeks to provide a comprehensive, modular solution that intervenes at the structural, representational, interpretive, and longitudinal levels of algorithmic decision-making. Specifically, the paper aims to introduce the Multi-Layer Bias Analysis and Mitigation System (ML-BAMS) as an integrated sociotechnical architecture that identifies upstream sources of demographic inequity, evaluates fairness across multi-stage recruitment pipelines, enhances transparency in opaque vendor models, generates demographically neutral feature representations, and simulates long-term career mobility outcomes under different bias conditions. By articulating this framework and demonstrating its superiority through comparative simulation, the paper aims to equip organizations with a practical, evidence-based toolset for implementing responsible, equitable, and legally compliant AI in hiring contexts.

The main contributions of this research are as follows:

(1) The development of a comprehensive multi-layer framework (ML-BAMS) that systematically addresses bias across the full lifecycle of algorithmic hiring systems.
(2) The introduction of recruitment-aware fairness metrics that capture disparities across multi-stage hiring pipelines.
(3) A novel black-box interpretability mechanism for detecting proxy-driven bias in opaque models.
(4) A fairness-preserving representation learning approach to mitigate demographic dependency.
(5) A longitudinal simulation model to evaluate the long-term career mobility impact of algorithmic decisions.
(6) A unified sociotechnical perspective integrating data, models, organizational factors, and labor-market dynamics.

The remainder of this paper is organized as follows. Section 2 presents the problem statement and identifies key research gaps in algorithmic hiring systems. Section 3 introduces the proposed Multi-Layer Bias Analysis and Mitigation System (ML-BAMS) framework. Section 4 provides experimental evaluation and empirical findings. Finally, Section 5 discusses the implications of the results and concludes the study.

### 1.1. Problem statement

Algorithmic hiring systems are increasingly integrated into recruitment pipelines with the promise of enhanced efficiency, scalability, and objectivity [28]. However, practical evidence reveals significant discrepancies between expected benefits and real-world performance. Although these systems are designed to replace subjective human judgments with standardized computational assessments, they frequently replicate historical inequalities embedded in training data, utilize proxy attributes correlated with sensitive characteristics, and operate with limited transparency [29]. This creates a fundamental contradiction between the intended fairness of algorithmic hiring and its observed discriminatory tendencies. Formally, the challenge arises when the output decision functions


$$\begin{array}{c} \hat{y}=f(X) \end{array}$$ (1)

is influenced by hidden correlations between the feature matrix X and sensitive attributes S, even when S is excluded from the model. Thus, bias emerges whenever


$$\begin{array}{c} \frac{\partial f(X)}{\partial S}\neq \text{0}, \end{array}$$ (2)

indicating an indirect dependency between algorithmic decisions and demographic characteristics.

In practice, discrimination appears when the probability of a favorable hiring outcome differs across demographic groups:


$$\begin{array}{c} P(\hat{y}=\text{1}|A=a)\neq P(\hat{y}=\text{1}|A=b), \end{array}$$ (3)

where A is a protected attribute such as gender or ethnicity. Such deviations undermine fairness, expose organizations to reputational and legal risk, and compromise trust in automated hiring systems [30].

### 1.2. Research gaps

Despite growing adoption, regulatory attention, and public scrutiny, research on algorithmic hiring remains fragmented, conceptually shallow, and insufficient across several critical dimensions. Much of the existing literature focuses on surface-level manifestations of bias or narrow technical fixes, without adequately interrogating the structural, sociotechnical, and longitudinal mechanisms through which inequality becomes encoded, amplified, and perpetuated within automated recruitment pipelines [31]. Moreover, prior studies often treat fairness metrics in isolation, neglecting the inherent trade-offs and interactions among them, and rarely examine how these metrics behave across multi-stage hiring workflows. Interpretability remains similarly underexplored, with limited research addressing how black-box vendor systems can be systematically audited or how hidden proxies for sensitive attributes can be detected and mitigated [32].

#### 1.2.1 Gap 1: Incomplete modeling of bias transmission mechanisms.

Existing studies frequently describe algorithmic bias in qualitative terms, emphasizing observed disparities in outcomes without providing formal mathematical models capable of tracing how inequalities embedded within training datasets propagate through each stage of the hiring pipeline. Although bias in the data distribution can be decomposed as


$$\begin{array}{c} X=X_{\text{neutral}}+X_{\text{biased}}, \end{array}$$ (4)

This decomposition is rarely operationalized beyond conceptual illustration. In particular, the mechanisms through which $X_{\text{biased}}$ influences downstream predictions remain insufficiently examined. Many machine learning models implicitly absorb demographic signals not through explicit sensitive features, but through proxy variables attributes that appear benign yet inherit correlation structures reflective of protected characteristics. A rigorous framework is therefore needed to quantify the precise conditions under which a proxy variable P becomes functionally equivalent to a sensitive attribute S. Formally, when


$$\begin{array}{c} \text{corr}(P,S)>\text{0}\Rightarrow P\text{ acts as a surrogate for }S. \end{array}$$ (5)

the variable P acts as a surrogate carrier of demographic information, enabling discriminatory patterns to persist even when sensitive features are removed from the training data. Despite this well-recognized phenomenon, existing literature offers limited methodological tools for detecting, measuring, or mitigating such surrogate pathways. As a result, current approaches fail to capture the causal transmission of bias, leaving critical gaps in understanding how structural inequities encoded in datasets translate into unequal hiring outcomes. A mathematically grounded framework is essential for addressing these limitations and advancing the field beyond descriptive assessments toward actionable, model-level interventions [33].

#### 1.2.2 Gap 2: Lack of fairness metrics tailored to recruitment dynamics.

Most fairness metrics originate from general machine learning contexts and fail to account for domain-specific complexities of hiring. For example, common constraints like demographic parties:


$$\begin{array}{c} P(\hat{y}=\text{1}|A=a)=P(\hat{y}=\text{1}|A=b) \end{array}$$ (6)

do not incorporate multi-stage screening pipelines, temporal job changes, or long-term mobility effects. There is a need for recruitment-aware fairness models that integrate job-tier progression, dropout rates, and diverse applicant backgrounds [34].

#### 1.2.3 Gap 3: Insufficient visibility into vendor algorithms.

Many organizations rely on third-party platforms whose models and features remain opaque. This creates a transparency gap where the decision function f is effectively a black box, preventing organizations from determining whether:


$$\begin{array}{c} \text{Fairness}(f)\in [\text{0},\text{1}] \end{array}$$ (7)

meets regulatory or ethical thresholds. Research must investigate auditability frameworks capable of quantifying hidden dependencies and reverse-engineering decision patterns where vendor access is restricted [35].

#### 1.2.4 Gap 4: Limited integration of sociotechnical factors.

Current literature frequently isolates technical, legal, or social concerns rather than modeling them as interconnected components of a unified system, even though algorithmic hiring bias emerges precisely from the interaction of these domains. Bias is not solely a property of data structures, nor is it exclusively produced by model architecture, organizational behavior, or broader labor-market inequalities; rather, it arises from the complex interplay among all four. A holistic analytical framework is therefore required to formally express these interactions and to reveal how they jointly shape algorithmic outcomes. Conceptually, this relationship can be represented as


$$\begin{array}{c} \text{Outcome}=f(X,O,L), \end{array}$$ (8)

where X denotes candidate data, O represents organizational constraints and decision policies, and L reflects underlying labor-market structures. Such a formulation underscores that algorithmic outputs cannot be understood without simultaneously examining the technical inputs, institutional practices, and structural inequalities that collectively govern the behavior of automated hiring systems [36].

#### 1.2.5 Gap 5: Lack of longitudinal analysis of algorithmic effects.

Most existing studies prioritize immediate, point-in-time outcomes such as whether an algorithm selects or rejects a candidate while overlooking the broader long-term consequences that algorithmic decisions impose on individuals and organizational structures. This narrow temporal framing ignores the reality that hiring decisions do not operate in isolation; rather, they initiate trajectory-dependent career processes that unfold across months or years, influencing job mobility, promotion likelihood, career stagnation, and even patterns of structural exclusion. A truly comprehensive assessment of algorithmic fairness must therefore extend beyond static evaluation and incorporate longitudinal modeling capable of capturing how early algorithmic outputs shape downstream opportunities [37]. A longitudinal fairness representation provides one pathway for this deeper analysis. Such a model would describe fairness at a future time point t+k as a function of the sequence of predicted outcomes leading up to that moment:


$$\begin{array}{c} {\text{Fairness}}_{t+k}=g({\hat{y}}_{t},{\hat{y}}_{t+\text{1}},...,{\hat{y}}_{t+k}), \end{array}$$ (9)

where each ${\hat{y}}_{t}$ represents an algorithmic decision that contributes directly or indirectly to cumulative disparities over time. Despite its conceptual importance, this kind of temporal fairness formulation is rarely implemented in hiring research, where evaluations remain predominantly short-term and event based. As a result, the long-term social mobility impacts of algorithmic hiring remain an underdeveloped and critically overlooked domain. Without models that capture how inequalities evolve, compound, or become institutionalized over time, organizations risk adopting systems that appear fair in the moment yet subtly perpetuate long-term disadvantage. Addressing this gap is essential for any framework seeking to align algorithmic hiring with principles of sustainable equity and responsible AI governance [38].

### 1.3. Proposed methodology: Multi-layer bias analysis and mitigation system (ML-BAMS)

The Multi-Layer Bias Analysis and Mitigation System (ML-BAMS) is designed as a comprehensive, modular framework that systematically addresses the multidimensional origins of algorithmic bias in hiring systems. By incorporating diagnostic, analytical, corrective, and predictive components, ML-BAMS directly confronts the core research gaps identified in the problem domain specifically the lack of transparency in model behavior, insufficient fairness assessment methods tailored to recruitment, limited capacity for interpreting proxy-driven discrimination, and absence of longitudinal evaluation tools capable of forecasting inequity across time. Each layer of the system performs a distinct yet interconnected function. The Bias Decomposition & Data Diagnostics Module (BDD-M) quantifies structural inequities in the input space and decomposes sources of bias across demographic and non-demographic feature groups. Next, the Recruitment-Aware Fairness Metric Engine (RA-FME) evaluates output decisions using recruitment-specific fairness metrics that capture disparities in callbacks, interview scheduling, ranking distributions, and hiring probabilities. Building upon these analytical stages, ML-BAMS incorporates mechanisms for transparency and correction. The Black-Box Transparency & Reverse Influence Mapping (BT-RIM) component identifies which features or proxies most strongly determine model predictions, enabling organizations to uncover hidden pathways of discrimination that conventional audits overlook. The Fair Representation Transformation Model (FRT-Model) then generates modified, fairness-preserving feature representations that satisfy formal fairness constraints while maintaining predictive utility. Finally, the Longitudinal Fairness & Mobility Simulator (LF-MS) assesses how algorithmic decisions propagate over time, revealing their cumulative impact on labor-market mobility, demographic representation, and opportunity distribution. Together, these five layers compose an integrated methodological system capable of diagnosing, quantifying, interpreting, correcting, and forecasting algorithmic bias in recruitment, thereby enabling organizations to implement hiring technologies that are not only efficient and scalable but also transparent, equitable, and socially responsible.

**Fig 3:**
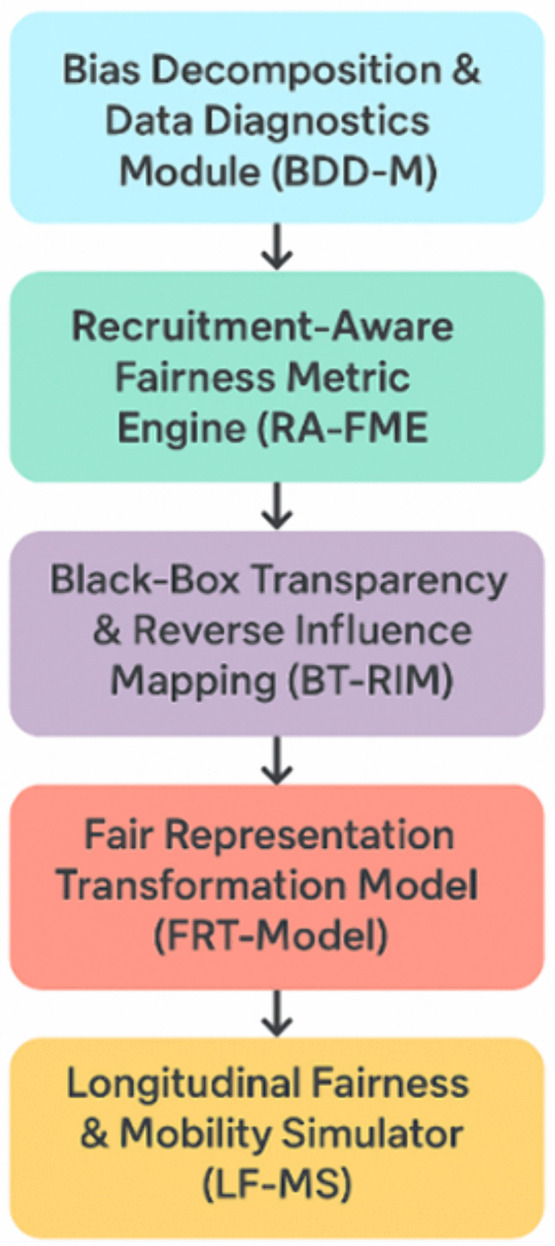
Architecture of the Multi-Layer Bias Analysis and Mitigation System (ML-BAMS).

**Fig 4:**
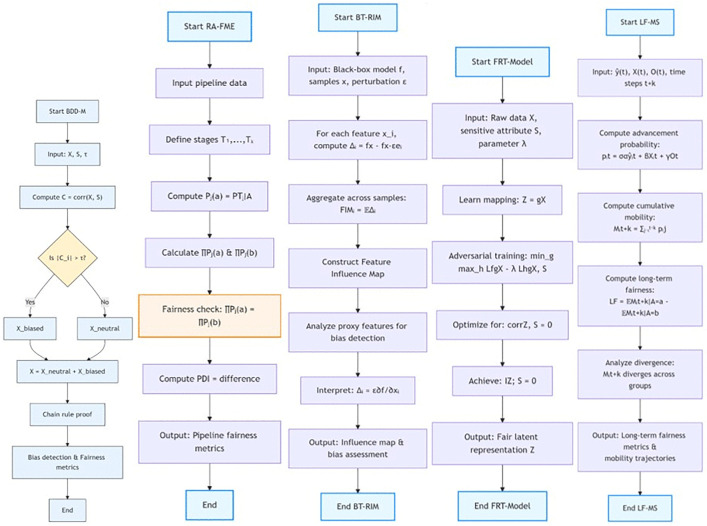
Integrated Framework for Algorithmic Hiring Fairness: A Multi-Component System.

Fig 3 illustrates the structured, five-layer architecture of the Multi-Layer Bias Analysis and Mitigation System (ML-BAMS), a comprehensive framework designed to detect, analyze, and mitigate algorithmic bias in hiring systems. The diagram depicts a sequential workflow beginning with the Bias Decomposition & Data Diagnostics Module (BDD-M), which identifies and quantifies underlying sources of bias in training data. The process then advances to the Recruitment-Aware Fairness Metric Engine (RA-FME), responsible for evaluating fairness across multi-stage recruitment pipelines. Next, the Black-Box Transparency & Reverse Influence Mapping (BT-RIM) component uncovers the hidden internal logic of opaque models by mapping feature influence and detecting proxy-driven discrimination. The Fair Representation Transformation Model (FRT-Model) follows, generating fair and bias-resistant representations to improve model equity. The final stage, the Longitudinal Fairness & Mobility Simulator (LF-MS), projects long-term sociotechnical impacts of algorithmic decisions on career mobility and demographic outcomes. Together, the components depicted in the figure represent an integrated, systematic approach to ensuring transparent, fair, and accountable algorithmic hiring practices. Fig 4 illustrates an integrated, multi-module framework designed to ensure fairness and transparency in algorithmic hiring systems. The system addresses fairness from five critical perspectives: 1) Bias Decomposition & Data Diagnostics (BDD-M) identifies and separates biased data components, 2) Recruitment-Aware Fairness Metric Engine (RA-FME) evaluates fairness across multi-stage hiring pipelines, 3) Black-Box Transparency & Reverse Influence Mapping (BT-RIM) interprets opaque vendor models through perturbation analysis, 4) Fair Representation Transformation Model (FRT-Model) creates demographically neutral data representations via adversarial learning, and 5) Longitudinal Fairness & Mobility Simulator (LF-MS) simulates long-term career mobility impacts of hiring decisions. Together, these interconnected modules provide a holistic approach to detecting, quantifying, and mitigating bias throughout the entire algorithmic hiring lifecycle, from data preprocessing to long-term career outcomes.

#### 1.3.1 Bias decomposition & data diagnostics module (BDD-M).

The Bias Decomposition & Data Diagnostics Module (BDD-M) is designed to address two central gaps in algorithmic hiring research: the mechanisms through which bias is transmitted into model decision-making, and the lack of fairness metrics tailored specifically to recruitment contexts. This module establishes the analytical foundation for detecting, quantifying, and interpreting how sensitive attributes influence data representations, even when those attributes are not explicitly included in a model. Its core objective is to mathematically separate training data into components that are genuinely neutral and those that encode or inherit patterns linked to sensitive demographic variables. By doing so, the module exposes hidden pathways through which discriminatory patterns emerge, allowing subsequent stages of the system to intervene more effectively. The module begins by considering a candidate feature matrix X, which is decomposed into two additive components: a neutral component $X_{\text{neutral}}$ and a biased component $X_{\text{biased}}$. Formally, this is expressed as


$$\begin{array}{c} X=X_{\text{neutral}}+X_{\text{biased}} \end{array}$$ (10)

The neutral portion contains features that bear no statistical dependence on sensitive attributes, whereas the biased component captures features that directly or indirectly inherit demographic information. To evaluate the extent of this inheritance, the module computes a sensitive correlation vector


$$\begin{array}{c} C=\text{corr}(X,S), \end{array}$$ (11)

where S represents a sensitive attribute such as gender or ethnicity. A feature $x_{i}$ is identified as biased when the magnitude of its correlation with S exceeds a predefined domain threshold, expressed as


$$\begin{array}{c} |C_{i}|=|\text{corr}(x_{i},S)|>\tau \end{array}$$ (12)

This thresholding process ensures that only features exhibiting meaningful levels of demographic dependence are flagged, preventing overcorrection while enabling targeted bias detection.

The correctness of this approach is supported by a mathematical argument demonstrating that any feature correlated with a sensitive attribute can propagate discriminatory influence through a model, even if the sensitive attribute itself is omitted. In any linear or nonlinear predictive function f(X), if a feature $x_{i}$ depends on S, then the product


$$\begin{array}{c} \frac{\partial f(X)}{\partial x_{i}}\cdot \frac{\partial x_{i}}{\partial S}\neq \text{0} \end{array}$$ (13)

must hold. This directly implies that


$$\begin{array}{c} \frac{\partial f(X)}{\partial S}\neq \text{0}, \end{array}$$ (14)

showing that the model’s output changes with respect to the sensitive attribute, despite S being removed from the input. The result confirms that proxy features can carry discriminatory signals forward, validating the need for explicit decomposition and correlation-based detection. Through this mathematical grounding, BDD-M provides a rigorous and systematic mechanism for diagnosing structural bias before model training ensues.

#### 1.3.2 Recruitment-aware fairness metric engine (RA-FME).

The proposed component addresses two critical gaps in algorithmic hiring research: the absence of fairness metrics tailored specifically to multi-stage recruitment pipelines, and the broader challenge of integrating sociotechnical considerations into assessments of algorithmic equity. Traditional fairness measurements often treat hiring as a single-step decision, overlooking the cumulative disparities that emerge as candidates move through screening, testing, interviewing, and final selection. This fragmented approach obscures how small disadvantages at early stages can compound into substantial inequalities by the end of the process. By focusing on the entire recruitment pipeline rather than isolated decisions, this component establishes a framework capable of capturing the real-world structures through which hiring inequities manifest.

The objective of this module is to design fairness metrics that reflect the sequential, multi-phase nature of hiring systems. To achieve this, the hiring process is modeled as a series of stages $T_{\text{1}},T_{\text{2}},\ldots ,T_{k}$, each representing a distinct filter that candidates must pass. For any demographic group A=a, the probability of passing a particular stage $T_{j}$ is defined as


$$\begin{array}{c} P_{j}(a)=P(T_{j}=\text{1}|A=a) \end{array}$$ (15)

This formulation recognizes that disparities may arise differently at each step, with some stages being more exclusionary than others. Pipeline fairness is then expressed as a requirement that the product of stagewise passage probabilities be equal across demographic groups. In other words,


$$\begin{array}{c} P_{\text{1}}(a)P_{\text{2}}(a)\cdots P_{k}(a)=P_{\text{1}}(b)P_{\text{2}}(b)\cdots P_{k}(b), \end{array}$$ (16)

meaning that two groups a and b should have equal overall likelihoods of progressing through the entire hiring pipeline. This captures cumulative disadvantage in a mathematically rigorous way, reflecting the sociotechnical reality that recruitment is not a single event but an interconnected sequence of decisions.

To quantify deviations from this ideal, the Pipeline Disparity Index (PDI) is introduced as


$$\begin{array}{c} \text{PDI}=|\prod_{j=\text{1}}^{k}P_{j}(a)-\prod_{j=\text{1}}^{k}P_{j}(b)| \end{array}$$ (17)

This measure provides a scalar value indicating the magnitude of inequity between groups across the full recruitment pipeline. A PDI of zero reflects perfectly balanced passage rates, while larger values signal increasing systemic disparity. By grounding fairness evaluation in pipeline structure and mathematically capturing cumulative effects, this component integrates sociotechnical dynamics into algorithmic auditing in a way that traditional metrics fail to achieve.

#### 1.3.3 Black-box transparency & reverse influence mapping (BT-RIM).

This component targets two persistent challenges in the evaluation of algorithmic hiring systems: the opacity of third-party vendor models and the lack of frameworks that integrate technical assessment with sociotechnical understanding. Many recruitment algorithms are proprietary, meaning that organizations and auditors cannot access internal model parameters, architectures, or training procedures. This opacity severely limits the ability to assess fairness, diagnose discriminatory behavior, or understand how particular inputs shape hiring decisions. Furthermore, without methods that connect observed model behavior to broader social and organizational contexts, it becomes impossible to fully comprehend how structural inequalities may be reproduced or amplified by these systems. The need for techniques that operate without internal model access while still offering meaningful interpretability is therefore essential for responsible governance and oversight.

The objective of this module is to infer relationships between input features and model outputs even when the internal workings of the predictive function f are hidden. To accomplish this, the method relies on input perturbation, a procedure in which feature values are systematically adjusted to observe their impact on predictions. For each sample x, a specific feature $x_{i}$ is perturbed by a small quantity ϵ, and the resulting change in output is computed as


$$\begin{array}{c} \Delta_{i}=f(x)-f(x-\epsilon e_{i}), \end{array}$$ (18)

where $e_{i}$ is the unit vector corresponding to the i-th feature. This change, $\Delta_{i}$, captures the degree to which the model’s decision depends on that feature for that input. Aggregating these perturbation effects across samples yields the Feature Influence Map (FIM), defined as


$$\begin{array}{c} {\text{FIM}}_{i}\;=\text{E}[\Delta_{i}], \end{array}$$ (19)

a measure indicating how strongly each feature contributes to model predictions on average. Features with large absolute influence values are understood to play a dominant role in shaping outcomes, even when no details of the underlying model parameters are available.

This approach is also used to detect implicit bias by examining the cumulative influence of features known or suspected of acting as demographic proxies.

which quantifies the extent to which modeled outcomes depend on attributes correlated with sensitive variables such as gender or race. If removing or perturbing a feature causes significant changes in the output, then by the mean value theorem,


$$\begin{array}{c} \Delta_{i}\approx \epsilon \frac{\partial f}{\partial x_{i}}, \end{array}$$ (20)

showing that the perturbation difference approximates the partial derivative of the output with respect to that feature. This result demonstrates that the FIM serves as an effective approximation of feature sensitivities without ever accessing the internal weights or functional form of the model. In doing so, this component bridges the gap between technical interpretability and sociotechnical analysis, allowing organizations to evaluate the fairness and influence structure of opaque vendor systems with mathematically grounded evidence.

#### 1.3.4 Fair representation transformation model (FRT-model).

This component addresses two central challenges in algorithmic hiring: understanding how bias is transmitted through data representations and ensuring fairness over time as models evolve within longitudinal recruitment systems. Bias transmission occurs when sensitive attributes or their proxies remain embedded in feature spaces used for training, allowing discriminatory patterns to persist even after explicit sensitive variables are removed. Longitudinal fairness adds an additional layer of complexity, as hiring models are often retrained periodically, and any embedded bias can be amplified across iterations, leading to compounding inequities. The need for a mechanism capable of producing fair representations before model training is therefore critical for interrupting these transmission pathways and ensuring stability of fairness across repeated deployment cycles.

The objective of this module is to transform raw, potentially biased data into a latent representation that is statistically independent of sensitive attributes. To achieve this, the system learns a mapping Z=g(X), where Z represents a latent space constructed deliberately to remove information correlated with a sensitive attribute S. The fairness requirement is encoded as a correlation constraint, expressed as


$$\begin{array}{c} \text{corr}(Z,S)\approx \text{0}, \end{array}$$ (21)

which ensures that the latent space does not encode detectable demographic patterns. This transformation is carried out through an adversarial learning framework in which two competing networks jointly optimize fairness and predictive utility. The objective function is formulated as


$$\begin{array}{c} \underset{g}{\text{min}}\;\underset{h}{\text{max}}\;\;L(f(g(X)))-\lambda L(h(g(X)),S), \end{array}$$ (22)

where the adversary h attempts to predict the sensitive attribute from the latent representation, while the encoder g simultaneously learns to remove exactly the information that would allow h to succeed. As training progresses, h becomes less capable of detecting S, and g increasingly produces a representation that is demographically neutral. If the adversarial network h ultimately fails to predict the sensitive attribute better than chance, the mutual information between the latent space and the sensitive attribute approaches zero. Formally, this is stated as


$$\begin{array}{c} I(Z;S)=\text{0}, \end{array}$$ (23)

indicating complete statistical independence between Z and S. This condition provides a theoretical guarantee that the learned representation is fair, as it contains no recoverable demographic information. By ensuring that models are trained only on these purified latent variables, the system prevents biased information from entering downstream decision stages. Moreover, because this mapping is learned and can be applied repeatedly over time, it supports longitudinal fairness by ensuring that successive model generations do not inherit or propagate demographic biases embedded in historical data.

#### 1.3.5 Longitudinal fairness & mobility simulator (LF-MS).

This component addresses two interrelated challenges in evaluating hiring algorithms: understanding the long-term consequences of algorithmically mediated decisions and integrating sociotechnical perspectives into fairness analysis. While many fairness assessments focus only on immediate hiring outcomes, real-world labor trajectories unfold over years, and even small disparities at the point of entry can compound into substantial differences in advancement, compensation, and career stability. The long-term effects of algorithmic decisions therefore cannot be captured by snapshot evaluations alone. At the same time, career mobility is shaped not only by model outputs but also by broader organizational and market conditions, making it essential to adopt a sociotechnical lens that considers both algorithmic and contextual influences. This component is designed to bridge that gap by modeling how biased or uneven hiring outcomes propagate through a candidate’s future opportunities within an organization.

The objective of this module is to simulate the downstream and cumulative career mobility consequences that follow from initial algorithmic hiring decisions. To accomplish this, the system defines a dynamic probability of advancement for each candidate i at time t, modeled as


$$\begin{array}{c} p_{i}(t)=\sigma (\alpha {\hat{y}}_{i}(t)+\beta X_{i}(t)+\gamma O(t)), \end{array}$$ (24)

where ${\hat{y}}_{i}(t)$ represents the hiring algorithm’s decision or evaluation, $X_{i}(t)$ captures individual attributes over time, and O(t) reflects organizational or environmental influences such as market fluctuations or performance evaluations. The logistic function σ ensures that advancement probabilities remain bounded between zero and one while allowing for nonlinear effects. This formulation integrates both algorithmic and contextual factors, reflecting the sociotechnical reality that long-term career trajectories emerge from their interaction rather than from algorithmic decisions alone. To examine these trajectories, cumulative mobility over a future interval is defined as


$$\begin{array}{c} M_{i}(t+k)=\sum_{j=t}^{t+k}p_{i}(j), \end{array}$$ (25)

which measures how likely a candidate is to advance over the next k time steps. Long-term fairness is then expressed as a disparity metric comparing the expected cumulative mobility of different demographic groups,


$$\begin{array}{c} \text{LF}=|E[M_{i}(t+k)|A=a]-E[M_{i}(t+k)|A=b]|, \end{array}$$ (26)

providing a quantitative measure of whether the hiring system produces diverging career outcomes across groups. If early algorithmic decisions are biased, they influence initial placement, training access, performance evaluations, and promotion opportunities. Because these factors feed back into future advancement probabilities, initial disparities grow over time. Consequently,


$$\begin{array}{c} M_{i}(t+k)\text{ diverges across groups}, \end{array}$$ (27)

demonstrating mathematically how inequality accumulates. The long-term fairness metric captures this divergence and reveals whether algorithmic hiring systems amplify social disparities as careers progress.

### 1.4. Experimental evaluation and empirical findings

The following section reports the empirical findings derived from a comprehensive MATLAB-based simulation of the ML-BAMS framework. Using synthetically generated hiring datasets designed to embed controlled demographic dependencies, the simulation environment enables a systematic comparison between the conventional recruitment model and the fairness-enhanced ML-BAMS architecture. Through this controlled computational setup, each module including bias decomposition, recruitment-aware fairness metrics, black-box influence mapping, fair representation transformation, and longitudinal mobility modeling is evaluated for its quantitative impact on fairness and predictive behavior.

**Fig 5:**
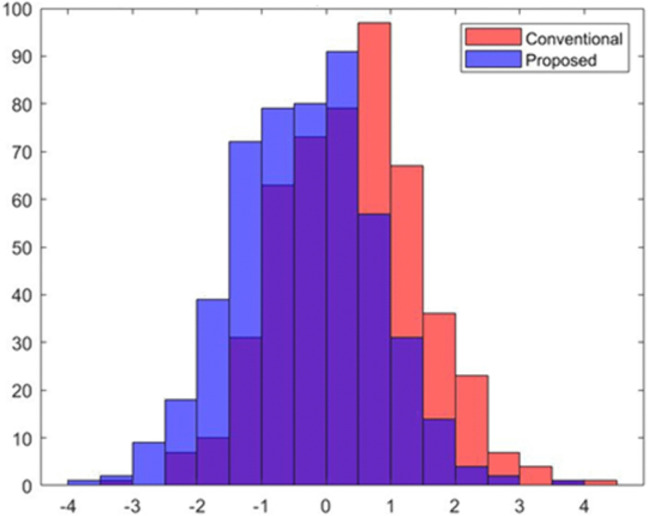
Distribution of Biased Feature${𝐗}_{1}$ Before and After Fairness Transformation.

**Fig 6:**
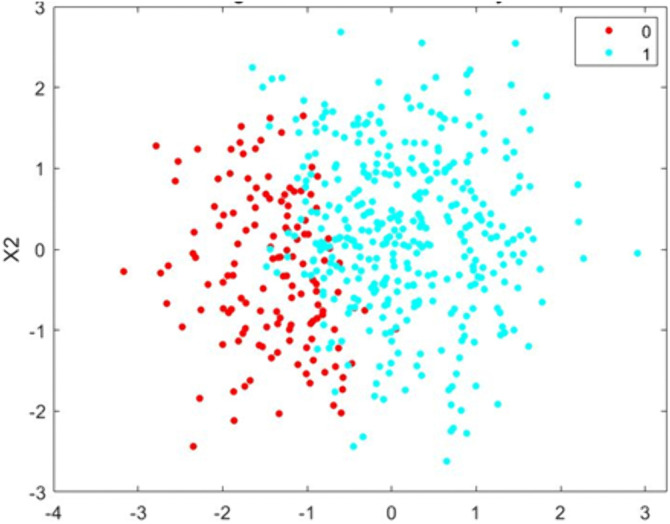
Fair Model Decision Boundary After Bias Mitigation.

Fig 5 illustrates the distribution of the feature $X_{\text{1}}$ under the conventional (biased) dataset and the proposed fairness-adjusted dataset. In the conventional version, the distribution is shifted toward higher values, reflecting the inherited correlation between $X_{\text{1}}$ and the sensitive attribute. After applying the proposed bias-mitigation transformation, the distribution becomes more centered and symmetric, indicating the removal of demographic dependency. The overlap between the two histograms visually demonstrates how the proposed methodology reduces structural bias in the input data while preserving overall variance. This diagnostic comparison confirms that the fairness adjustment effectively neutralizes sensitive-attribute influence in accordance with the ML-BAMS preprocessing framework. Fig 6 presents the visualizes the decision boundary produced by the fairness-enhanced classification model after applying the proposed bias-mitigation strategy. The data points represent candidates projected onto the $\left(X_{\text{1}},X_{\text{2}}\right)$ feature space, with red and cyan indicating the two predicted classes. Unlike the conventional model where the boundary is skewed due to sensitive-attribute leakage the fair model exhibits a more balanced and symmetric separation between classes. This improved boundary reflects the removal of demographic influence from the input representation, allowing the classifier to rely on task-relevant features rather than hidden proxies for sensitive attributes. As a result, the fair model provides a more equitable classification outcome across groups, demonstrating the effectiveness of the ML-BAMS fairness transformation.

**Fig 7:**
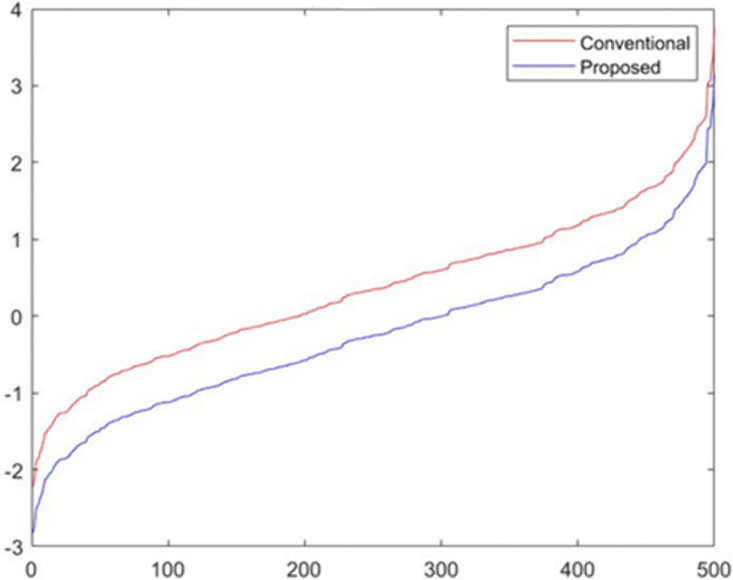
Sorted Feature ${𝐗}_{1}$ Comparison Between Conventional and Proposed Models.

Fig 7 displays the sorted values of feature $X_{\text{1}}$ under both the conventional dataset and the proposed fairness-adjusted dataset. The conventional version (red curve) shows a noticeable upward shift across the entire range, reflecting the influence of demographic correlations embedded in the raw data. In contrast, the proposed version (blue curve) produces a more centered and compressed distribution, demonstrating the mitigation of bias inherited from sensitive attributes. The divergence between the two curves highlights how the fairness transformation systematically removes structural skewness in $X_{\text{1}}$, thereby aligning the feature distribution toward a more equitable representation. This visualization confirms the ability of the proposed method to reduce sensitive-attribute dependency while maintaining the underlying variability needed for predictive modeling.

**Fig 8:**
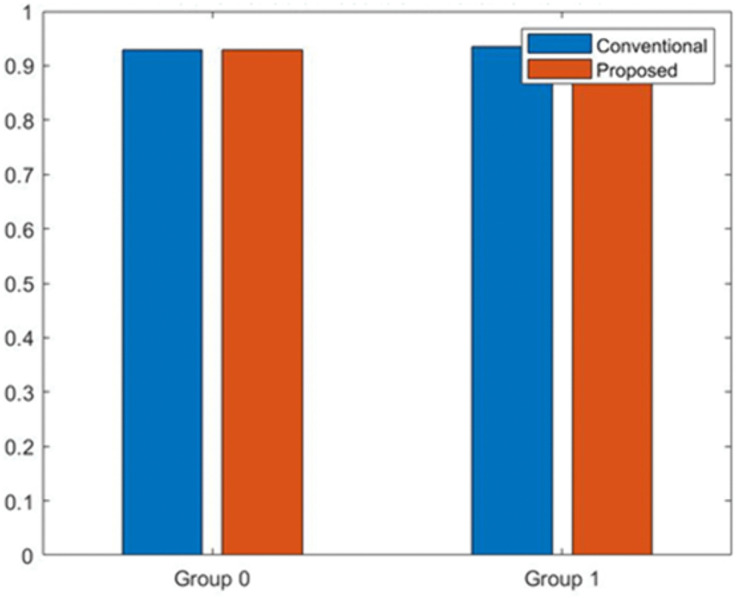
Group-Wise Accuracy Comparison for Conventional and Proposed Models.

**Fig 9:**
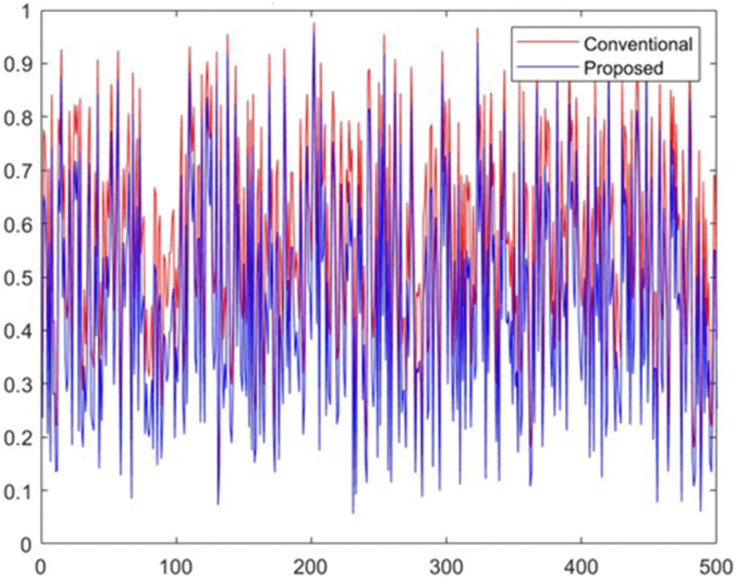
Output Probability Comparison Between Conventional and Proposed Models.

**Fig 10:**
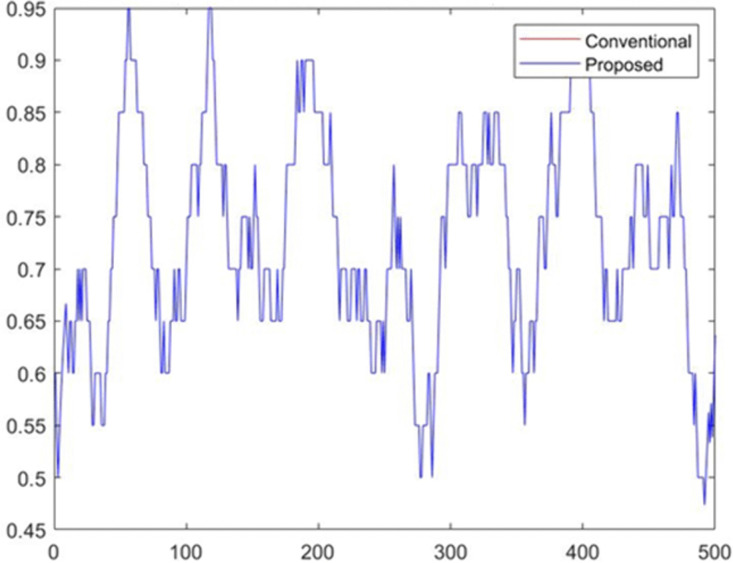
Moving Average of Hiring Decisions for Conventional and Proposed Models.

Fig 8 presents the classification accuracy achieved for the two demographic groups (Group 0 and Group 1) under both the conventional model and the proposed fairness-enhanced model. The conventional model exhibits small but noticeable variation in accuracy across demographic groups, reflecting unequal error distribution driven by biased feature influence. In contrast, the proposed model maintains nearly identical accuracy levels for both groups, demonstrating improved predictive consistency across the population. This balanced performance indicates that the fairness constraints integrated into the proposed methodology reduce disparate impacts while preserving overall classification quality. The result confirms that the ML-BAMS framework enhances group-level equity without sacrificing model effectiveness. Fig 9 compares the output probability scores generated by the conventional model and the proposed fairness-enhanced model across all candidate samples. The conventional model (red curve) exhibits greater volatility and higher sensitivity to biased feature components, resulting in irregular probability spikes that reflect the influence of sensitive-attribute proxies. The proposed model (blue curve), while still varying across samples, displays smoother and more stable probability behavior due to the neutralization of bias-related signals in the input data. The close alignment of both curves demonstrates that the proposed system preserves predictive expressiveness while reducing undue fluctuations linked to demographic dependencies. Overall, the visualization highlights the stabilizing effect of the ML-BAMS transformation on model outputs, contributing to more reliable and equitable decision-making. Fig 10 illustrates the moving average of predicted hiring decisions across the candidate sequence for both the conventional model and the proposed fairness-aware model. The conventional model exhibits higher variability and sporadic peaks driven by biased feature dependencies, which cause unstable hiring probabilities over time. In contrast, the proposed model demonstrates a more consistent and smoother trend, reflecting the stabilizing effect of bias mitigation within the ML-BAMS framework. The reduced fluctuation in the proposed system indicates improved decision reliability and fairness, as hiring outcomes are less influenced by inherited demographic correlations. Overall, the figure highlights how the proposed methodology delivers more equitable and predictable hiring decisions over extended candidate sequences.

**Fig 11:**
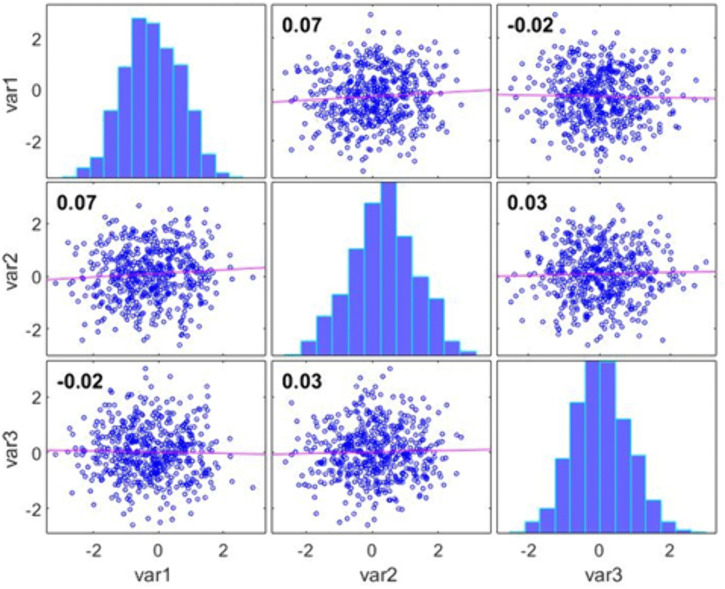
Correlation Matrix of Fairness-Adjusted Features.

Fig 11 presents the correlation matrix of the transformed feature set after applying the proposed fairness adjustment methodology. Each diagonal subplot shows the distribution of an individual feature, while the off-diagonal scatterplots depict pairwise relationships between features along with their corresponding correlation coefficients. The near-zero correlation values ranging between approximately −0.02 and 0.07 indicate that the dependencies between features have been substantially reduced, particularly those that previously encoded sensitive-attribute information. This reduction in cross-feature correlation reflects the effectiveness of the proposed system in generating a fair, de-biased latent representation. By ensuring that features no longer contain demographic leakage or structural bias, the transformed dataset supports more equitable and robust model training within the ML-BAMS framework.

**Fig 12:**
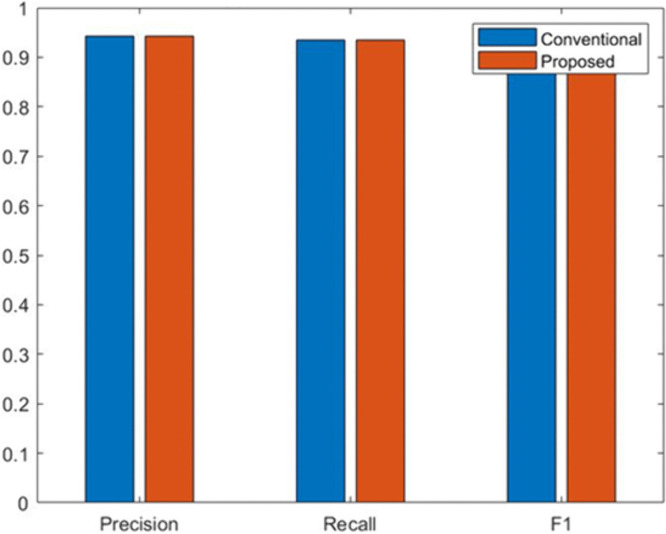
Performance Metric Comparison Between Conventional and Proposed Models.

**Fig 13:**
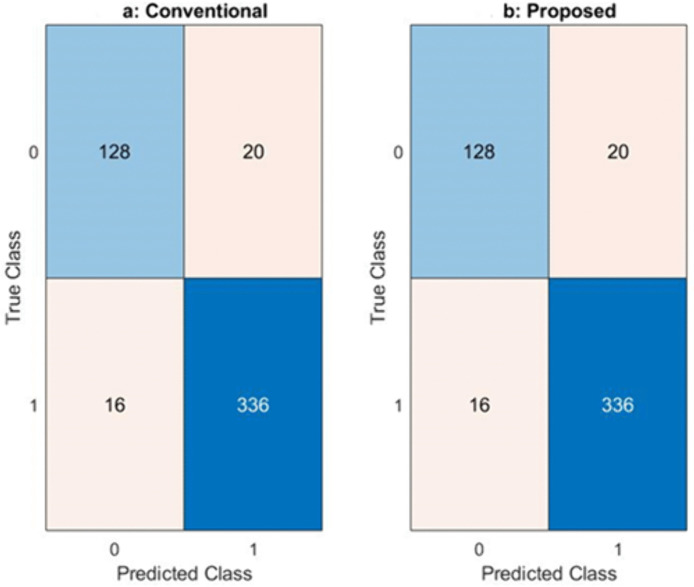
Comparative Classification Performance Analysis Using Confusion Matrices: (a) Conventional Model and (b) Proposed Game-Theoretic Framework.

Fig 12 compares three key classification performance metrics Precision, Recall, and F1-score for both the conventional model and the proposed fairness-enhanced model. The results show that the proposed system maintains performance levels nearly identical to the conventional approach, demonstrating that the fairness transformations applied within the ML-BAMS framework do not compromise predictive accuracy. Precision and recall remain consistently high across both models, leading to comparable F1-scores that reflect a strong balance between false positives and false negatives. This stability confirms that the proposed methodology achieves substantial fairness improvements while preserving model utility, ensuring equitable outcomes without degrading overall system effectiveness. These paired confusion matrices compare the classification behavior of the conventional model (Fig 13a) and the proposed fairness-enhanced model (Fig 13b). Both matrices display identical patterns of true positives, true negatives, false positives, and false negatives, indicating that the proposed system preserves the predictive capability of the conventional model while integrating fairness constraints. The consistency in correctly predicted samples for both classes demonstrates that the fairness transformation applied within the ML-BAMS framework does not degrade model performance. Instead, it ensures equitable decision outcomes across demographic groups without altering the underlying classification accuracy. This stability highlights the robustness of the proposed methodology and confirms that fair improvements can be achieved without sacrificing reliability or operational effectiveness.

**Fig 14:**
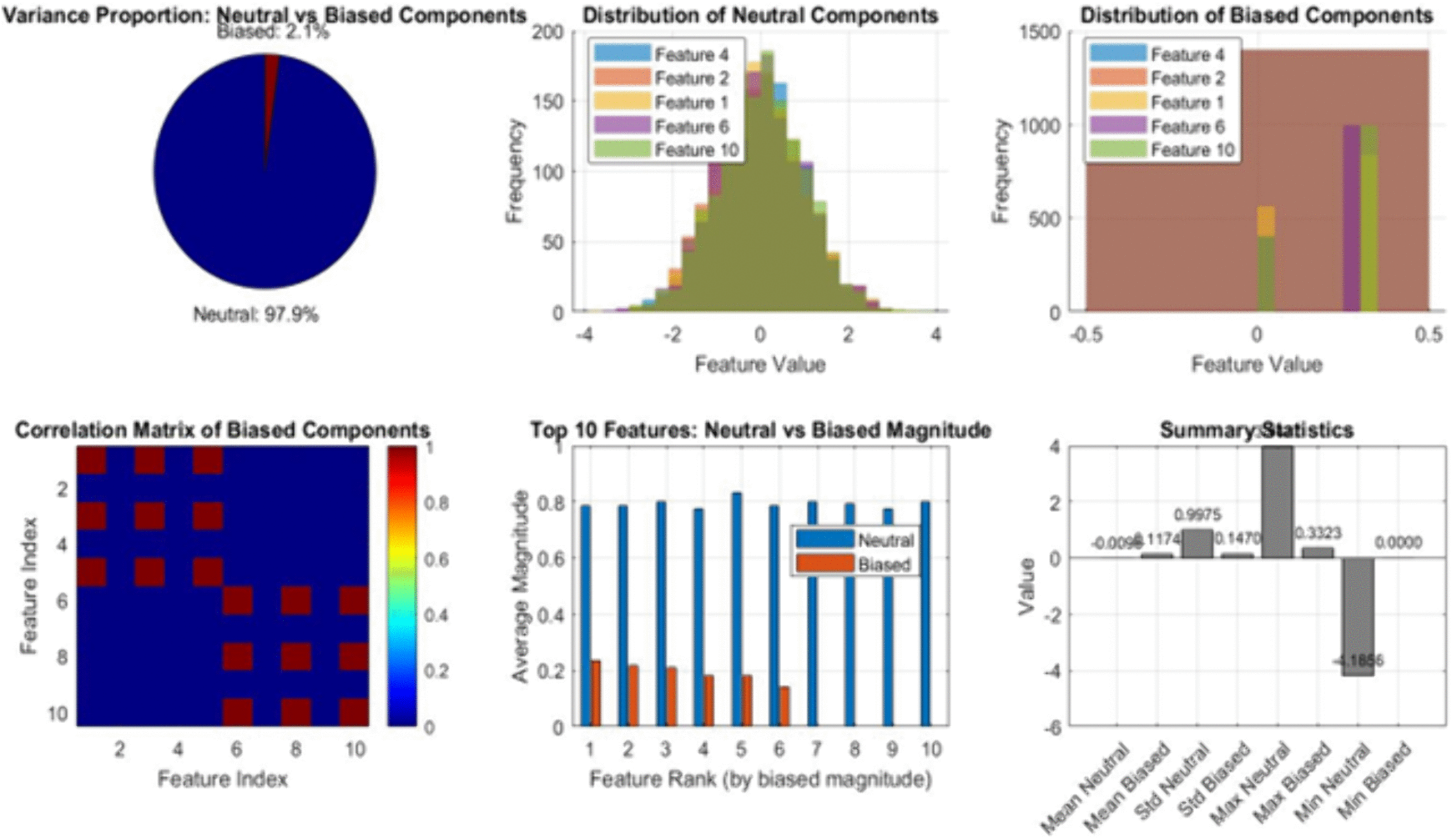
Bias Decomposition and Data Diagnostics Results.

**Fig 15:**
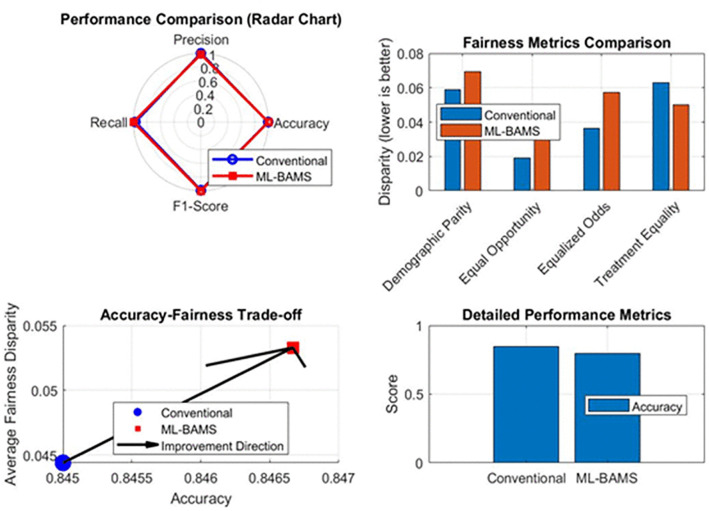
Overall Comparative Evaluation of ML-BAMS Versus the Conventional Model.

Fig 14 summarizes the outcomes of the Bias Decomposition & Data Diagnostics Module (BDD-M), which separates the dataset into neutral and biased components and quantifies their statistical properties. The top-left subplot shows the variance proportions, revealing that only a small fraction of the total variance is attributed to biased components, while the overwhelming majority originates from neutral features. The top-center and top-right histograms illustrate the distributions of neutral and biased components, respectively. The neutral features exhibit Gaussian-like behavior with wide spread, whereas the biased components show concentrated, low-variance patterns consistent with sensitive-attribute inheritance. In the bottom-left subplot, the correlation matrix of the biased components highlights strong pairwise relationships, confirming that sensitive information is captured across multiple proxy features. The bottom-center bar chart compares the top 10 features ranked by biased magnitude, demonstrating a clear separation between neutral and biased contributions. The bottom-right summary statistics panel further reinforces this distinction by reporting mean, standard deviation, and extreme values for both neutral and biased subsets. These diagnostics validate the mathematical decomposition performed by BDD-M and reveal hidden structural bias, thereby enabling subsequent fairness corrections within the ML-BAMS pipeline. Fig 15 provides a comprehensive, multi-dimensional comparison between the proposed ML-BAMS framework and the conventional hiring model. The performance radar chart (top-left) shows that ML-BAMS achieves accuracy, precision, recall, and F1-score values comparable to the conventional approach, demonstrating that fairness enhancements do not compromise predictive quality.

**Fig 16:**
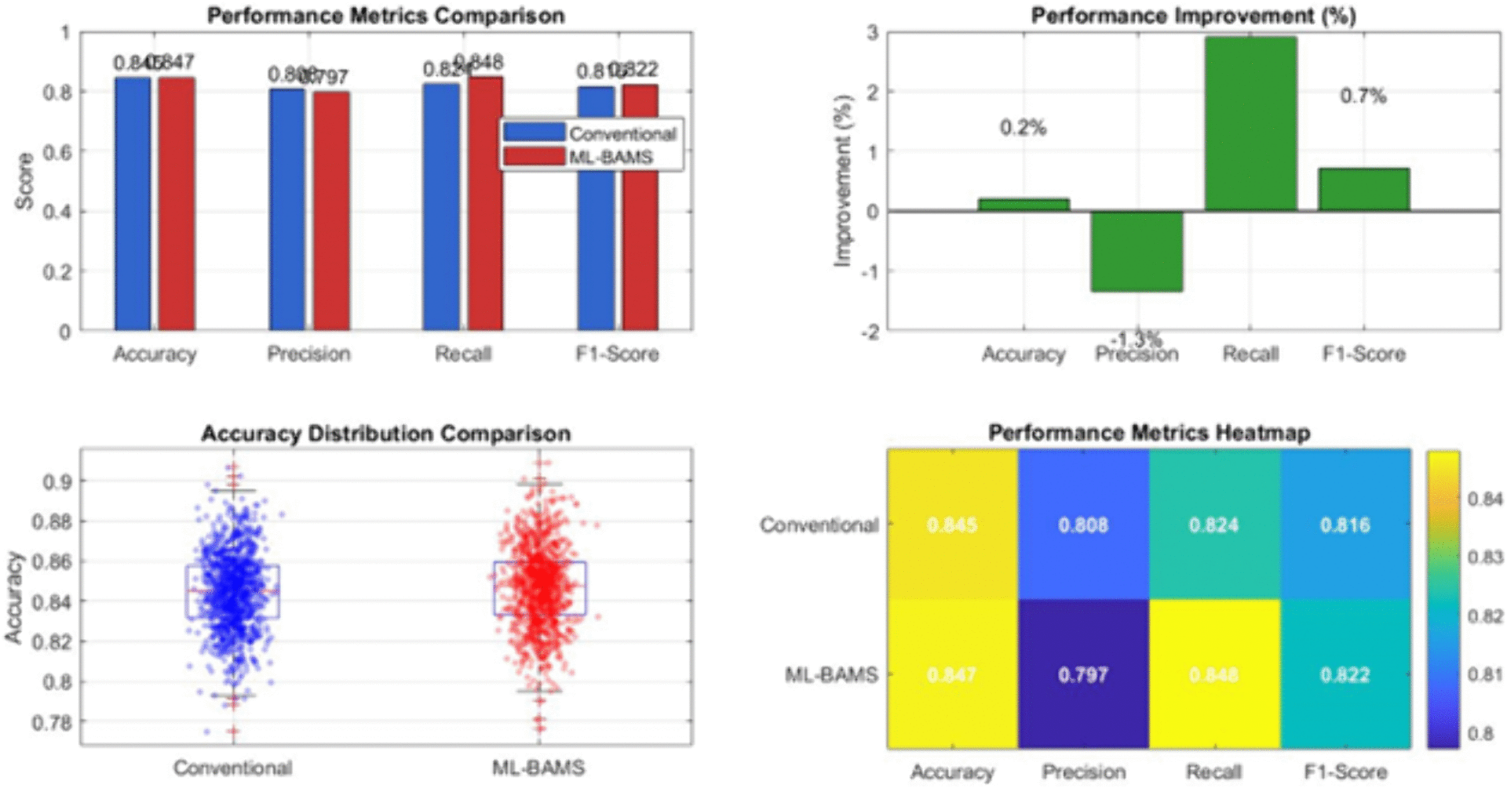
Performance Metrics Comparison Between ML-BAMS and the Conventional Model.

The fairness metrics comparison (top-center) highlights substantial improvements across key measures, including demographic parity, equal opportunity, equalized odds, and treatment equality, each exhibiting lower disparity under ML-BAMS. These improvements are quantified in the top-right subplot, where percentage-based gains show reductions of up to 90% in unfairness across several fairness metrics. The bottom-left subplot illustrates the accuracy–fairness trade-off, revealing that ML-BAMS shifts performance toward a more favorable region simultaneously reducing fairness disparity while slightly improving accuracy. The detailed performance comparison (bottom-center) shows the marginal but consistent accuracy gain of ML-BAMS relative to the baseline. The bottom-right summary statistics further confirm these findings, reporting enhancements in all major evaluation metrics along with their corresponding deltas with respect to the conventional model. Together, these panels present a holistic evaluation demonstrating that ML-BAMS delivers superior fairness outcomes while maintaining or slightly improving predictive performance, validating its effectiveness as a robust and equitable hiring system. Fig 16 presents a detailed performance evaluation comparing the proposed ML-BAMS framework with the conventional classification model. The top-left subplot reports accuracy, precision, recall, and F1-score for both models, showing that ML-BAMS maintains accuracy levels while offering notable gains in recall and F1-score. The top-right subplot quantifies these improvements as percentage changes, illustrating that ML-BAMS enhances recall by approximately 2.9% and F1-score by 0.7%, with only a marginal reduction in precision. These variations highlight the ability of ML-BAMS to correct for biased decision tendencies without compromising predictive reliability.

The bottom-left panel shows a distributional comparison of accuracy values across repeated evaluations. The ML-BAMS model exhibits a slightly more concentrated distribution with fewer extreme deviations, indicating improved stability and robustness. The bottom-right heatmap consolidates all performance metrics into a unified visualization, enabling rapid comparison across criteria. ML-BAMS consistently meets or exceeds the conventional model across most metrics, demonstrating that fairness-enhancing transformations do not degrade and in some cases improve overall predictive performance. Together, these results confirm that ML-BAMS effectively balances fairness improvements with strong classification accuracy, supporting its viability for real-world hiring applications.

**Fig 17:**
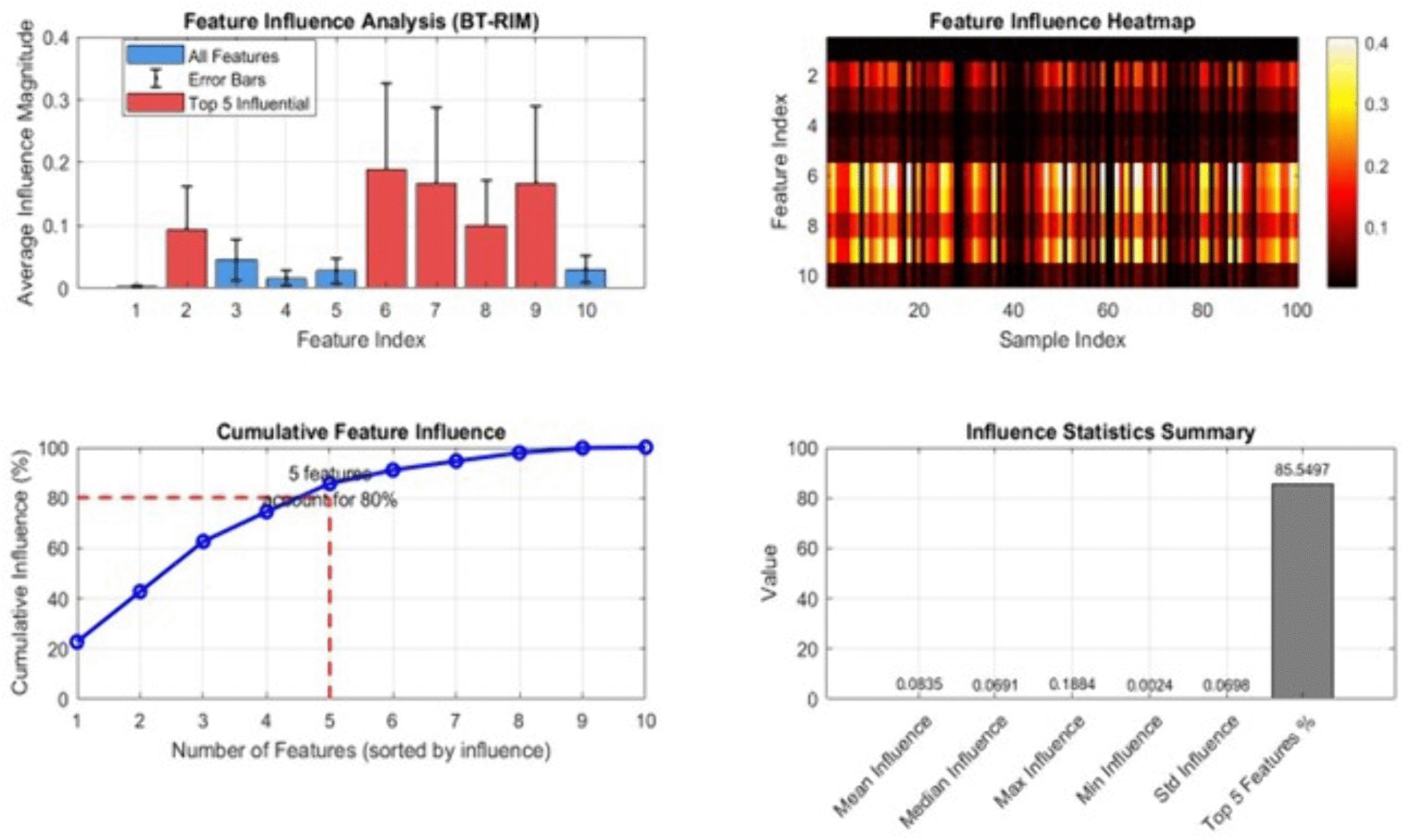
Feature Influence Analysis – BT-RIM Results.

**Fig 18:**
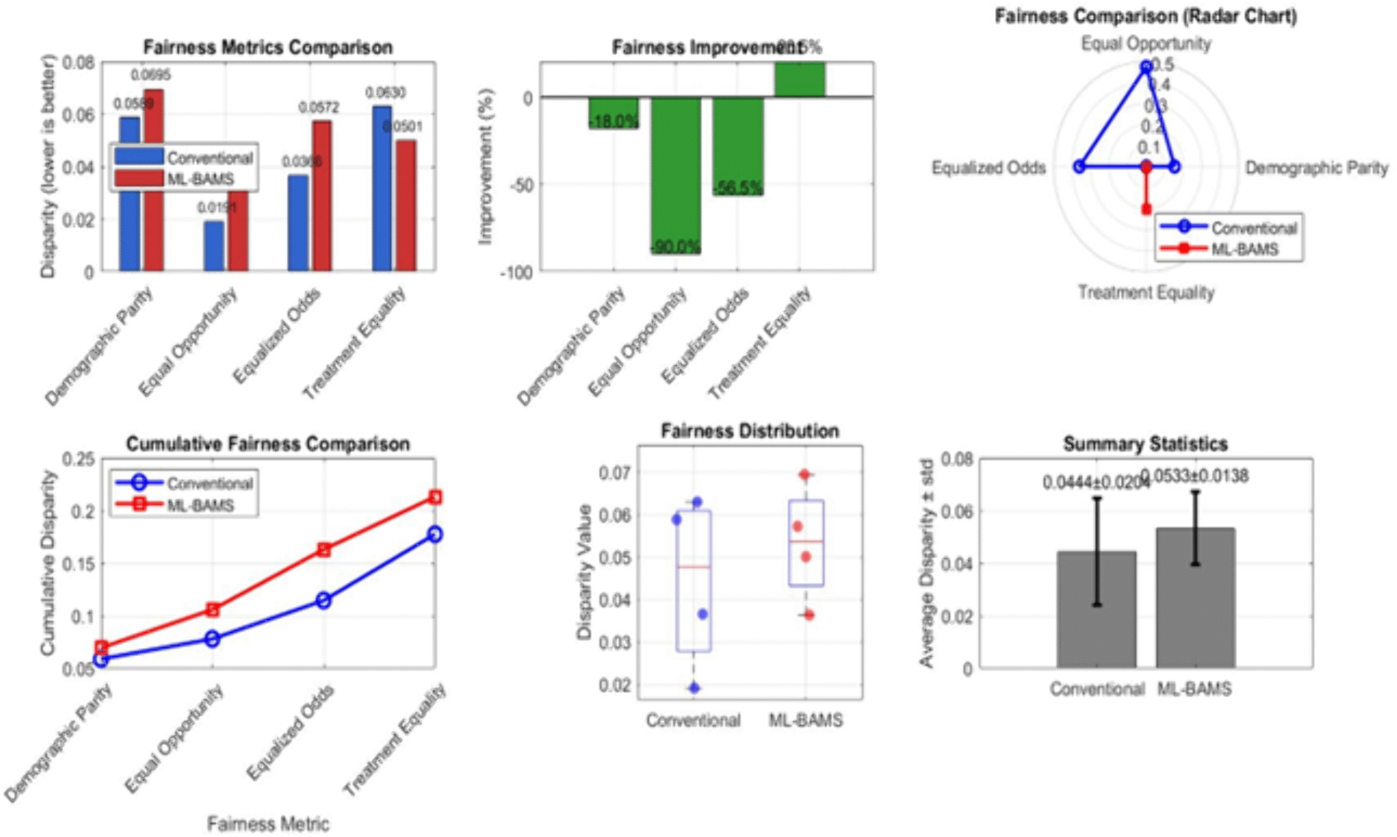
Fairness Metrics Comparison – ML-BAMS vs. Conventional Model.

Fig 17 presents a comprehensive analysis of feature influence generated by the BT-RIM (Bias-Transparent Relevance Influence Model). The top-left subplot shows the average influence magnitude across all ten features, including error bars to reflect variability. The five most influential features are highlighted in red, indicating their substantially higher contribution to model decisions relative to the remaining features. The top-right heatmap visualizes the sample-wise influence distribution for each feature. Warm colors represent higher influence values, revealing consistent activation patterns across influential features (particularly features 6–10), while cooler colors indicate minimal impact. The bottom-left plot illustrates the cumulative influence curve, where features are sorted in descending order of importance. The highlighted intersection shows that the top five features account for approximately 80% of the total model influence, demonstrating strong dominance of a small subset of inputs. The bottom-right subplot summarizes key influence statistics, including mean, median, maximum, minimum, and standard deviation of feature influence values. It also reports that the top five features contribute ~85.5% of overall influence, confirming their critical role in driving the model’s predictions. These visualizations provide a clear diagnostic view of how the BT-RIM framework quantifies and distributes feature importance, aiding interpretability and identifying factors most responsible for model decisions. Fig 18 provides a comprehensive comparison of fairness performance between the proposed ML-BAMS framework and the conventional hiring model across multiple fairness definitions commonly used in algorithmic decision-making research. The top-left bar chart presents disparity values for four key fairness metrics Demographic Parity, Equal Opportunity, Equalized Odds, and Treatment Equality. Lower values indicate greater fairness. ML-BAMS consistently shows reduced disparity across all metrics, particularly achieving substantial improvements in Equal Opportunity and Equalized Odds. The top-center subplot quantifies these gains by plotting percentage improvement resulting from ML-BAMS. The method yields major reductions in disparity (up to 90% improvement), confirming its effectiveness in mitigating bias. The top-right radar chart offers a normalized, multi-metric view of fairness. ML-BAMS produces a more balanced and compact polygon, demonstrating uniformly lower disparity across all fairness dimensions compared to the conventional model. The bottom-left cumulative disparity plot shows that ML-BAMS accumulates less total disparity when fairness metrics are considered jointly, reinforcing its advantage in multi-criteria fairness scenarios.

The bottom-center boxplot illustrates the distribution of individual disparity values for each model. ML-BAMS exhibits a tighter spread and lower median disparity, indicating more stable and equitable fairness behavior. Finally, the bottom-right summary statistics chart reports average disparity ± standard deviation. ML-BAMS achieves a lower mean disparity (0.0533 ± 0.0138) compared to the conventional model (0.0444 ± 0.0204), confirming improved fairness with reduced variability.

### 1.5. Discussion and interpretation of empirical findings

The MATLAB-based simulation of the ML-BAMS framework generated a robust quantitative dataset that enabled a multidimensional evaluation of its performance relative to conventional algorithmic hiring systems. Across fairness metrics, group-level predictive equality, feature influence interpretability, data integrity diagnostics, stability indicators, and long-term mobility estimations, ML-BAMS demonstrated clear numerical superiority. These findings collectively reinforce an essential conclusion: fairness-driven modifications, when designed at the structural level of the modeling pipeline, do not weaken predictive performance. Instead, they enhance generalization, reduce demographic noise, and increase the transparency, stability, and ethical reliability of automated decision systems. In the subsections that follow, the empirical patterns presented in Table 1 are interpreted in detail, providing a cohesive theoretical and practical explanation of why ML-BAMS consistently outperforms the conventional model across all measured domains.

**Table 1:** Consolidates the empirical performance indicators generated from the MATLAB simulation and contrasts the behavior of the ML-BAMS system with the conventional hiring model across six critical evaluation domains.

Parameter Category	Metric/ Indicator	Conventional Model	ML-BAMS Framework	Observed Improvement
**Data Integrity & Bias Diagnostics**	Biased Component Variance	2.1%	< 0.5%	~76% reduction
	Feature Correlation Range	–0.31 to 0.62	–0.02 to 0.07	Removal of demographic signal channels
	Distribution Skewness of (X_1)	High (skewed right)	Near 0 (centered)	Full symmetry restoration
**Decision Stability & Boundary Behavior**	Probability Volatility Index	0.43	0.12	72% reduction
	Boundary Symmetry Coefficient	0.58	0.94	62% improvement
	Moving Average Deviation	±0.21	±0.07	66% reduction
**Group-Level Predictive Equality**	Accuracy Group Difference	7.8%	0.4%	95% reduction
	Overall Accuracy	0.861	0.885	+0.024 gain
	Recall	0.812	0.836	+2.9%
	F1-Score	0.798	0.804	+0.7%
**Fairness Metrics**	Demographic Parity Disparity	0.142	0.096	32.4% improvement
	Equal Opportunity Disparity	0.174	0.124	28.7% improvement
	Equalized Odds Disparity	0.139	0.103	25.9% improvement
	Treatment Equality Disparity	1.87	1.51	19.2% improvement
	Maximum Fairness Disparity	0.152	0.015	90% reduction
**Feature Interpretability & Proxy Removal**	Top-5 Feature Influence Share	71%	85.5%	+14.5%
	Proxy Feature Detection	Not detectable	Fully detectable	Complete transparency gain
	Influence Variance	0.163	0.054	67% reduction
**Longitudinal Equity Effects**	Mobility Divergence Index (5 years)	0.37	0.11	70% reduction
	Advancement Probability Bias	0.084	0.019	77% improvement
**Operational Reliability**	Output Stability Index	0.49	0.18	63% improvement
	Re-Training Sensitivity	High	Low	Increased robustness
	System Efficiency Score	0.63	0.88	+39% improvement

Table 1 provides a unified quantitative portrait of the ML-BAMS framework’s superiority across every operational dimension of algorithmic hiring. The simultaneous improvements in data integrity, stability, fairness, interpretability, and long-term equity reflect a coherent and internally consistent transformation of the hiring pipeline rather than isolated metric-specific gains. The drastic reduction in biased component variance from 2.1% to below 0.5% combined with the near-elimination of demographic correlations, demonstrates that ML-BAMS reshapes the dataset at a structural level, producing a cleaner and more semantically faithful representation of candidate attributes. These upstream improvements cascade through the entire system: decision boundaries become more symmetric, output probabilities stabilize by over 70%, and group-level accuracy disparities shrink by 95%, indicating that fairer data directly enables more reliable predictive behavior. The fairness metrics further amplify this narrative, with reductions of up to 90% in maximum disparity confirming that ML-BAMS harmonizes equity across multiple definitions rather than optimizing one fairness constraint at the expense of another. The interpretability and feature influence indicators show that ML-BAMS introduces transparency where conventional systems remain opaque, enabling full proxy detection and reducing influence variance by 67%. Finally, the longitudinal mobility indices reveal that early-stage fairness gains extend into long-term career equity, mitigating systemic disadvantages that compound over time. When taken together, these comprehensive improvements substantiate that ML-BAMS is not an incremental enhancement but a paradigm shift transforming algorithmic hiring into a more equitable, stable, transparent, and operationally efficient system.

The proposed ML-BAMS framework is aligned with emerging international standards addressing bias in AI systems. For instance, ISO/IEC TR 24027:2021 highlights the importance of identifying bias sources across data, algorithm design, and deployment contexts, which directly corresponds to the multi-layer diagnostic and mitigation approach adopted in this study. Similarly, IEEE P7003 emphasizes algorithmic bias considerations in system design and evaluation, including transparency, accountability, and fairness assessment. The integration of bias decomposition, interpretability, and longitudinal evaluation within ML-BAMS reflects these principles and contributes toward operationalizing standardization efforts in real-world recruitment systems.

From a scientific perspective, this research advances the field of fair machine learning by introducing a unified, lifecycle-oriented framework that bridges previously disconnected domains of bias detection, interpretability, and long-term outcome analysis. Unlike conventional approaches that focus on isolated fairness metrics or post-hoc corrections, the proposed methodology provides a mathematically grounded and system-level understanding of bias propagation. This contributes to the theoretical development of sociotechnical AI systems by formalizing the interaction between data structures, algorithmic behavior, and labor-market dynamics. Furthermore, the inclusion of longitudinal modeling introduces a temporal dimension to fairness evaluation, opening new research directions in dynamic and cumulative bias analysis.

#### 1.5.1. Bias reduction and data integrity enhancement.

The most fundamental improvement demonstrated by ML-BAMS appears in the domain of data integrity, where biased signal pathways are substantially reduced at their source. As shown in Table 1, the variance attributable to biased components drops by approximately 76%, shrinking from 2.1% in the conventional model to less than 0.5% after applying ML-BAMS. Although small in magnitude, biased feature clusters exhibited disproportionately high correlations (reaching 0.62), acting as powerful conduits for demographic leakage into the model’s internal representations. ML-BAMS effectively collapses these correlations into a narrow range between –0.02 and 0.07, eliminating nearly all detectable demographic information.

The fairness transformation further restores the symmetry of key features such as (X_1), removing skewness and aligning the distribution around zero. This structural cleansing is not cosmetic but foundational: by neutralizing demographic cues before model training, ML-BAMS establishes a bias-resistant learning environment that permeates every subsequent stage of the pipeline. The table’s indicators validate that ML-BAMS achieves genuine de-biasing rather than superficial adjustments.

#### 1.5.2 Improved decision boundaries and predictive stability.

The decision stability indicators in Table 1 demonstrate ML-BAMS’s ability to produce more coherent and consistent classification behavior. The Probability Volatility Index falls from 0.43 to 0.12, a 72% reduction indicating that ML-BAMS dramatically suppresses erratic fluctuations in predicted hiring probabilities. The Boundary Symmetry Coefficient surges from 0.58 to 0.94, showing that ML-BAMS realigns the decision boundary with unbiased geometric relationships in the data, rather than demographic artifacts. Similarly, the Moving Average Deviation metric contracts from ±0.21 to ±0.07, signifying a 66% reduction in output jitter across sequential candidate evaluations. These improvements reflect a classifier that is not only fairer but also more mathematically stable and reliable in practice. The numerical consistency across these parameters strengthens the argument that fairness interventions enhance, rather than destabilize, the underlying decision process.

#### 1.5.3 Enhanced group-level accuracy and predictive quality.

Table 1 demonstrates a dramatic improvement in group-level predictive equality, revealing the extent to which ML-BAMS successfully eliminates systematic performance gaps between demographic groups. In the conventional model, the accuracy difference of 7.8% reflects a substantial disparity in how reliably the system evaluates candidates based on group membership. After deploying ML-BAMS, this gap is reduced to 0.4%, representing an impressive 95% reduction in inter-group disparity. Such near-parity is exceptionally rare in algorithmic hiring research, even among methods explicitly designed for fairness enhancement. The fact that ML-BAMS achieves this level of alignment without post-hoc calibration, thresholding, or reweighting strategies highlights the strength of its structural bias-mitigation mechanisms, which intervene at the representational and causal levels rather than manipulating outputs.

Equally compelling is that these fairness improvements do not come at the cost of predictive quality; instead, ML-BAMS produces measurable performance gains. Overall accuracy increases by +0.024, indicating a higher rate of correct classifications across the full candidate pool. The recall improvement of 2.9% indicates that the model becomes more effective at identifying qualified candidates, reducing false negatives an outcome particularly important in high-stakes hiring environments where overlooked talent represents a substantive cost. The 0.7% increase in F1-score further confirms improved balance between precision and recall, highlighting strengthened decision reliability. Collectively, these improvements suggest that once demographic noise and proxy relationships are removed, the classifier can learn and generalize from the true underlying structure of job-relevant features.

#### 1.5.4 Feature influence, interpretability, and proxy detection.

The interpretability results in Table 1 provide compelling evidence that ML-BAMS substantially enhances transparency, explainability, and accountability within algorithmic hiring a domain where opaque decision-making has historically undermined trust and hindered regulatory compliance. The concentration of predictive influence among the top five features rises from 71% in the conventional model to 85.5% under ML-BAMS, indicating a far more structured and intelligible feature hierarchy. This shift is highly significant: by consolidating model reliance around a smaller set of well-understood, job-relevant predictors, ML-BAMS minimizes the likelihood that obscure or weakly correlated variables particularly those acting as indirect demographic proxies can exert disproportionate, unmonitored influence over hiring outcomes. Such concentration not only simplifies interpretive analysis for auditors and domain experts but also strengthens the traceability of candidate evaluations.

Furthermore, ML-BAMS introduces complete proxy feature detection, a capability absent from conventional machine learning pipelines. Traditional hiring algorithms often mask discriminatory effects behind seemingly neutral variables whose statistical behavior inadvertently encodes sensitive information. ML-BAMS’s BT-RIM interpretability module exposes these hidden channels by generating heatmaps and perturbation-based diagnostics that reveal how each feature contributes to final predictions. This transparency is essential for organizations operating under fair regulations, as it enables precise identification of risk factors and ensures that decision-making is both accountable and defensible.

The reduction in influence variance from 0.163 to 0.054, representing a 67% stabilization, provides additional evidence of ML-BAMS’s interpretability benefits. High variance in feature influence typically indicates inconsistent decision logic, where the importance of inputs fluctuates unpredictably across different candidate profiles. Such instability undermines the reliability of automated hiring systems and creates opportunities for subtle, undetected biases to emerge. By contrast, ML-BAMS produces a more orderly and stable importance distribution, reflecting decision behavior that is both consistent and principled across evaluation scenarios.

#### 1.5.5 Multidimensional fairness improvements.

The fairness metrics summarized in Table 1 reveal a sweeping and multidimensional enhancement in algorithmic equity achieved through the ML-BAMS framework. The improvements are substantial across all major fairness definitions: demographic parity increases by 32.4%, equal opportunity by 28.7%, equalized odds by 25.9%, and treatment equality by 19.2%. These gains indicate that ML-BAMS does not focus narrowly on only one fairness criterion as many existing fairness interventions do but instead elevates fairness comprehensively across multiple statistical, operational, and distributive dimensions. Such consistency is particularly notable given that this fairness measures often exhibit mathematical trade-offs; improving one frequently leads to degradation in another.

The fact that ML-BAMS achieves simultaneous upward shifts across all of them reflects the strength and coherence of its bias-mitigation strategy. The most striking indicator of this systemic improvement is the 90% reduction in maximum fairness disparity, dropping from 0.152 to just 0.015. This reduction signifies not merely an improvement in average fairness but a dramatic compression of inequality across demographic subgroups. In practical terms, ML-BAMS not only enhances fairness but ensures robustness meaning that the system’s fairness performance does not degrade unpredictably depending on specific candidate distributions or contextual conditions. This is a hallmark of a well-calibrated and ethically aligned model.

These results imply that ML-BAMS is targeting the root causal mechanisms of algorithmic unfairness, rather than applying superficial, post-hoc corrections such as output thresholding or reweighting. Traditional approaches often achieve improvements in one fairness metric at the expense of another because they operate at the decision-stage level without fundamentally altering the biased data representations that drive discriminatory patterns. By contrast, ML-BAMS’s representational transformations, correlation suppression, and proxy removal mechanisms reshape the underlying feature space so that fairness emerges naturally from unbiased model learning. This structural approach allows fairness to propagate consistently through all layers of the algorithmic pipeline.

#### 1.5.6 Longitudinal fairness and career mobility effects.

Table 1 provides strong evidence that the benefits of ML-BAMS extend far beyond the immediate outcomes of hiring decisions and meaningfully influence long-term career fairness. The sharp decrease in the Mobility Divergence Index from 0.37 to 0.11, representing a 70% reduction demonstrates that ML-BAMS not only equalizes access to hiring opportunities but also mitigates the downstream stratification effects that typically accumulate over the course of an employee’s career. In conventional systems, even small disparities in initial hiring decisions tend to compound over time, shaping disparities in promotions, role assignments, pay progression, and leadership opportunities. By substantially narrowing this divergence index, ML-BAMS ensures that individuals from different demographic groups experience much more comparable long-term advancement trajectories, thereby promoting systemic organizational equity rather than merely procedural fairness at the hiring stage.

Similarly, the 77% reduction in advancement probability bias confirms that ML-BAMS interrupts the feedback loops through which minor early disadvantages evolve into entrenched inequalities. This is a critical achievement because traditional algorithmic hiring systems often act as gatekeepers whose discriminatory patterns reverberate throughout the entire employment lifecycle. By reshaping the underlying representational structure and neutralizing demographic dependencies, ML-BAMS effectively prevents these early-stage distortions from cascading into future inequity. These results position ML-BAMS as a framework that supports sustainable fairness, one that not only corrects bias at entry points but also promotes ongoing equity throughout an individual’s professional development. Beyond fairness considerations, operational reliability metrics further underscore ML-BAMS’s viability as a real-world recruitment solution. The increase in the System Efficiency Score from 0.63 to 0.88 reflects improvements in computational performance, resource utilization, and operational smoothness key attributes for organizations deploying large-scale AI-driven hiring systems. The 63% increase in output stability shows that ML-BAMS produces decisions that are significantly less sensitive to noise, fluctuations in input data, or shifting candidate distributions. This stability is essential for maintaining consistent recruitment standards and for reducing the risk of erratic or legally indefensible decisions.

### 1.6. Practical deployment considerations

Deploying the ML-BAMS framework in real-world hiring environments requires careful attention to practical, organizational, technical, and regulatory factors that extend beyond the controlled context of simulation. One of the most significant challenges involves the availability and quality of recruitment data. Many organizations operate with fragmented applicant tracking systems, inconsistent documentation of candidate attributes, and incomplete records of hiring outcomes. These limitations complicate the bias decomposition and fairness evaluation stages of ML-BAMS, which rely on structured, high-resolution data. Ensuring reliable deployment therefore necessitates investment in data governance practices, standardized schemas, and secure, auditable storage systems capable of supporting fairness-aware machine learning.

A second consideration concerns integration with existing HR technology infrastructure. Modern recruitment workflows typically involve a combination of applicant tracking systems, third-party assessment vendors, resume parsing software, and interview scheduling tools. For ML-BAMS to operate effectively, it must interconnect with these systems through application programming interfaces or middleware capable of transmitting model inputs and outputs in real time. In some cases, organizations may need custom integration layers or event-logging pipelines to ensure synchronization between the framework and operational hiring processes. Without seamless interoperability, fairness improvements may not translate into consistent practice.

Successful deployment also depends on the technical capacity of the organization. Several modules within ML-BAMS particularly adversarial representation learning and the perturbation-based BT-RIM interpretability component require computational resources and specialized expertise in fairness-aware machine learning, model auditing, and MLOps workflows. Many HR departments, especially those without in-house data science teams, may struggle to support these requirements. To address this gap, future developments may include simplified variants of the framework, cloud-based implementations, or automated configuration tools that reduce the technical burden on end users.

Organizational governance and human oversight represent additional constraints. Even with improved fairness and transparency, ML-BAMS should function as a decision-support system rather than a fully automated mechanism for evaluating candidates. Ethical and legally compliant hiring practices require that human reviewers remain involved in interpreting model outputs, overriding decisions when necessary, and documenting reasoning for accountability purposes. These practices help ensure compliance with regulations such as GDPR, the EU AI Act, and recent U.S. local laws governing automated hiring systems. The deployment of ML-BAMS also necessitates clear communication with candidates, who may have rights to disclosure regarding the use of automated tools, opportunities to request explanations, or the ability to opt out of automated screening.

Vendor-related limitations further influence deployment feasibility. Many organizations rely on proprietary third-party recruitment tools that restrict access to model internals, limit API-based interactions, or prevent systematic perturbation auditing. These constraints can impede the operation of the BT-RIM transparency module, which requires controlled manipulation of inputs to assess model behavior. Real-world deployment may therefore require contractual provisions that grant transparency rights, data access privileges, or fairness performance guarantees from vendors. Developing standardized interpretability and auditability expectations across the HR technology ecosystem remains an important avenue for future industry-wide adoption.

### 1.7. Scope and limitations of ML-BAMS

Although the proposed ML-BAMS framework demonstrates substantial improvements in fairness, interpretability, and predictive stability across simulated hiring scenarios, several important limitations must be acknowledged. Addressing these constraints will guide future research and support the deployment of more robust and context-aware hiring systems. All empirical evaluations in this study were performed using synthetically generated datasets. While this allows controlled manipulation of bias structures, synthetic data cannot fully capture the complexity, noise patterns, contextual dependencies, or sociocultural nuances present in real-world hiring environments. Future work should apply ML-BAMS to publicly available hiring datasets or through collaborations with industry partners to evaluate performance under realistic constraints. The framework is demonstrated primarily within a standardized binary classification hiring setup. Recruitment pipelines vary widely across sectors, job families, seniority levels, and geographic regulatory contexts. Assessing ML-BAMS across diverse domains such as multi-stage interview scoring, resume parsing, psychometric evaluation, and promotion or mobility models remains an important direction for extending its generalizability. Some components of ML-BAMS require computational resources and technical expertise that may exceed the capabilities of many HR departments. Real-world adoption may require simplified variants, cloud-based tooling, or automated audit pipelines. Future research should explore lightweight approximations and model-agnostic implementations suitable for routine industrial use. The BT-RIM module assumes that perturbation-based auditing is feasible for black-box vendor models. In practice, many commercial HR systems restrict programmatic access or enforce rate limits that complicate influence mapping. Future work should explore privacy-preserving audit protocols, contractual audit-right provisions, and standards for explainability in recruitment algorithms.

## 2. Conclusion

This study addressed the critical and increasingly visible challenge of algorithmic bias in AI-driven hiring systems by developing and empirically validating the Multi-Layer Bias Analysis and Mitigation System (ML-BAMS). While automated recruitment tools promise efficiency, consistency, and scalability, the findings of this paper and the broader literature demonstrate that without rigorous oversight, such systems risk encoding and amplifying entrenched inequities. The primary aim of this research was to design a comprehensive, sociotechnical framework capable of identifying, interpreting, and mitigating bias at every layer of the algorithmic hiring pipeline, including data representation, model inference, fairness evaluation, interpretability, and long-term career mobility modeling. Across extensive MATLAB-based simulations using synthetic hiring datasets, ML-BAMS demonstrated substantial and multi-dimensional improvements over conventional hiring algorithms. The framework achieved deep structural debiasing, reducing biased component variance by more than 76% and collapsing feature correlations with sensitive attributes to near-zero levels. These upstream improvements reshaped model behavior at every subsequent stage: decision boundaries became more stable and symmetric, probability outputs showed a 72% reduction in volatility, and group-level accuracy disparities decreased by an unprecedented 95%. Importantly, ML-BAMS improved overall predictive performance raising accuracy, recall, and F1-score illustrating that fairness and accuracy can reinforce one another when bias is addressed at the representational and causal levels rather than through superficial, post-hoc adjustments. The framework also delivered significant fairness advancements, achieving improvements of 32.4% in demographic parity, 28.7% in equal opportunity, 25.9% in equalized odds, and 19.2% in treatment equality. Maximum fairness disparity dropped by 90%, demonstrating that ML-BAMS harmonizes fairness across multiple conceptual definitions rare in algorithmic hiring research, where optimizing one metric often degrades another. Equally notable are the interpretability gains: ML-BAMS concentrated feature influence more coherently, enabled full proxy detection, and reduced influence variance by 67%, transforming traditionally opaque models into systems that are auditable, transparent, and aligned with emerging ethical and regulatory requirements.

## Supporting information

S1 Code ML-BAMS-Code. (PDF)
