# Supplementary material for: Algorithmic bias in HR recruitment systems: A qualitative analysis of managerial risk and sociological implications
Source: PLoS One. 2026 Jun 3;21(6):e0349400. doi: 10.1371/journal.pone.0349400 (PMC13232804; doi:10.1371/journal.pone.0349400)
Supplement: S1 Code — (PDF) [file pone.0349400.s001.pdf]

Supplementary File
ML-BAMS-Code

1- **Create a MATLAB file entitled (run_all.m)**

```
clc; clear; close all;

current_folder = fileparts(mfilename('fullpath'));

modules_path = fullfile(current_folder, 'modules');

if exist(modules_path, 'dir')

   addpath(modules_path);

else

   error('Modules folder not found. Please ensure correct package structure.');

end

rng(42);

[X, S, y] = generate_data(500);

[biased_idx, corr_vals] = bias_decomposition(X, S);

X_fair = fair_representation(X, S);

[mdl_b, mdl_f, yb, yf] = train_models(X, X_fair, y);

metrics = fairness_metrics(y, yb, yf, S);

FIM = influence_mapping(X, mdl_b);

LF_gap = longitudinal_simulation(X, yf, S);

disp(metrics);

fprintf('Long-term fairness gap: %.4f\n', LF_gap);
```

2- **Create a folder entitled modules in the same folder of (run_all.m)**
3- **The modules folder contains the following MATLAB files (bias_decomposition.m, fair_representation.m, fairness_metrics.m, generate_data.m, influence_mapping.m, longitudinal_simulation.m, train_models.m)**
4- **The details of the files as follows:**

Supplementary File
ML-BAMS-Code

bias_decomposition.m

```
function [biased_idx,corr_vals]=bias_decomposition(X,S)

corr_vals=corr(X,S);

tau=0.1;

biased_idx=abs(corr_vals)>tau;

end
```

fair_representation.m

```
function Xf=fair_representation(X,S)

N=size(X,1); Xf=X;

for i=1:size(X,2)

 b=regress(X(:,i),[S ones(N,1)]);

 Xf(:,i)=X(:,i)-b(1)*S;

end

end
```

fairness_metrics.m

```
function m=fairness_metrics(y,yb,yf,S)

acc_b=[mean(yb(S==0)==y(S==0)) mean(yb(S==1)==y(S==1))];

acc_f=[mean(yf(S==0)==y(S==0)) mean(yf(S==1)==y(S==1))];

dp_b=abs(mean(yb(S==0))-mean(yb(S==1)));

dp_f=abs(mean(yf(S==0))-mean(yf(S==1)));

m=struct('Acc_Biased',acc_b,'Acc_Fair',acc_f,'DP_Biased',dp_b,'DP_Fair',dp_f);

end
```

Supplementary File
ML-BAMS-Code

generate_data.m

```
function [X,S,y]=generate_data(N)

S=randi([0,1],N,1);

X1=randn(N,1)+1.5*S;

X2=randn(N,1);

X=[X1 X2];

y=double(X1+X2+0.5*S>0);

end
```

influence_mapping.m

```
function FIM=influence_mapping(X,model)

eps=0.01; FIM=zeros(size(X,2),1);

for i=1:size(X,2)

 Xp=X; Xp(:,i)=X(:,i)-eps;

 y1=predict(model,X); y2=predict(model,Xp);

 FIM(i)=mean(abs(y1-y2));

end

end
```

longitudinal_simulation.m

```
function gap=longitudinal_simulation(X,y_pred,S)

T=10; N=size(X,1); M=zeros(N,1);

for t=1:T

 p=1./(1+exp(-(0.5*y_pred+0.3*X(:,1))));

 M=M+p;

end

gap=abs(mean(M(S==0))-mean(M(S==1)));

end
```

Supplementary File
ML-BAMS-Code

train_models.m

```
function [mb,mf,yb,yf]=train_models(X,Xf,y)

mb=fitcsvm(X,y,'KernelFunction','linear');

mf=fitcsvm(Xf,y,'KernelFunction','linear');

yb=predict(mb,X);

yf=predict(mf,Xf);

end
```

Supplementary File
ML-BAMS-Code

```
clear; clc; close all; rng(1);

 N = 500;

 % Sensitive attribute (0/1)

S = randi([0 1],N,1);

 % Features (biased)

X1 = randn(N,1) + 1.2*S;   % biased feature

X2 = randn(N,1);

X3 = randn(N,1);

 X = [X1 X2 X3];

 % Fair transformation (remove correlation with S)

X1_fair = X1 - mean(X1(S==1)) + mean(X1(S==0));

X_fair = [X1_fair X2 X3];

 scores_base = 0.7*X1 + 0.3*X2;

scores_fair = 0.7*X1_fair + 0.3*X2;

 prob_base = 1./(1+exp(-scores_base));

prob_fair = 1./(1+exp(-scores_fair));

 y_base = prob_base > 0.5;

y_fair = prob_fair > 0.5;

 figure;

histogram(X1,20,'FaceColor','r','FaceAlpha',0.5); hold on;

histogram(X1_fair,20,'FaceColor','b','FaceAlpha',0.5);

title('Distribution of Biased Feature X1');

legend('Conventional','Proposed');

 figure;

gscatter(X1_fair,X2,y_fair,'rb','..');

title('Fair Decision Boundary');
```

```
xlabel('X1'); ylabel('X2');
 figure;
plot(sort(X1),'r','LineWidth',2); hold on;
plot(sort(X1_fair),'b','LineWidth',2);
title('Sorted Feature X1');
legend('Conventional','Proposed');
 acc0 = mean(y_fair(S==0)==y_base(S==0));
acc1 = mean(y_fair(S==1)==y_base(S==1));
 figure;
bar([acc0 acc1; acc0 acc1]');
title('Group Accuracy');
legend('Conventional','Proposed');
 figure;
plot(prob_base,'r'); hold on;
plot(prob_fair,'b');
title(' Output Probability');
legend('Conventional','Proposed');
 window = 20;
mov_base = movmean(y_base,window);
mov_fair = movmean(y_fair,window);
 figure;
plot(mov_base,'r'); hold on;
plot(mov_fair,'b');
title('Moving Average Hiring Decisions');
legend('Conventional','Proposed');
 figure;
```

Supplementary File
ML-BAMS-Code

```
corrplot(X_fair);

title('Fair Correlation Matrix');

 precision = sum(y_fair==1 & y_base==1)/sum(y_fair==1);

recall = sum(y_fair==1 & y_base==1)/sum(y_base==1);

f1 = 2*(precision*recall)/(precision+recall);

 figure;

bar([precision recall f1]);

set(gca,'XTickLabel',{'Precision','Recall','F1'});

title(' Performance Metrics');

 figure;

confusionchart(y_base,y_fair);

title('Confusion Matrix');

 biased = abs(corr(X,S)) > 0.2;

neutral = ~biased;

 figure;

 subplot(2,2,1)

pie([sum(neutral) sum(biased)]);

title('Variance Proportion');

 subplot(2,2,2)

histogram(X(:,neutral));

title('Neutral Features');

 subplot(2,2,3)

histogram(X(:,biased));

title('Biased Features');

 subplot(2,2,4)

bar(abs(corr(X,S)));
```

```
title('Feature-Sensitive Correlation');

 sgtitle('Bias Decomposition Results');
```
